# Meningeal lymphatic drainage: novel insights into central nervous system disease

**DOI:** 10.1038/s41392-025-02177-z

**Published:** 2025-05-05

**Authors:** Qiang Zhang, Yin Niu, Yingpei Li, Chenyang Xia, Zhi Chen, Yujie Chen, Hua Feng

**Affiliations:** 1 Department of Neurosurgery and State Key Laboratory of Trauma, Burn and Combined Injury, Southwest Hospital, Third Military Medical University (Army Medical University), Chongqing, 400038 China; 2 Department of Neurosurgery, The 961st Hospital of the Chinese People’s Liberation Army Joint Logistic Support Force, Qiqihar Medical University, Qiqihar, 161000 Heilongjiang China; 3 Chongqing Key Laboratory of Intelligent Diagnosis, Treatment and Rehabilitation of Central Nervous System Injuries, Southwest Hospital, Third Military Medical University (Army Medical University), Chongqing, 400038 China; 4 Chongqing Clinical Research Center for Neurosurgery, Southwest Hospital, Third Military Medical University (Army Medical University), Chongqing, 400038 China; 5 CAS Key Laboratory of Separation Science for Analytical Chemistry, Dalian Institute of Chemical Physics, Chinese Academy of Sciences, Dalian, 116023 China

**Keywords:** Neurological disorders, Diseases of the nervous system

## Abstract

In recent years, increasing evidence has suggested that meningeal lymphatic drainage plays a significant role in central nervous system (CNS) diseases. Studies have indicated that CNS diseases and conditions associated with meningeal lymphatic drainage dysfunction include neurodegenerative diseases, stroke, infections, traumatic brain injury, tumors, functional cranial disorders, and hydrocephalus. However, the understanding of the regulatory and damage mechanisms of meningeal lymphatics under physiological and pathological conditions is currently limited. Given the importance of a profound understanding of the interplay between meningeal lymphatic drainage and CNS diseases, this review covers seven key aspects: the development and structure of meningeal lymphatic vessels, methods for observing meningeal lymphatics, the function of meningeal lymphatics, the molecular mechanisms of meningeal lymphatic injury, the relationships between meningeal lymphatic vessels and CNS diseases, potential regulatory mechanisms of meningeal lymphatics, and conclusions and outstanding questions. We will explore the relationship between the development, structure, and function of meningeal lymphatics, review current methods for observing meningeal lymphatic vessels in both animal models and humans, and identify unresolved key points in meningeal lymphatic research. The aim of this review is to provide new directions for future research and therapeutic strategies targeting meningeal lymphatics by critically analyzing recent advancements in the field, identifying gaps in current knowledge, and proposing innovative approaches to address these gaps.

## Introduction

Mammals possess two tubular circulatory systems: the blood circulatory system and the lymphatic circulatory system. The lymphatic system plays a crucial role in maintaining fluid balance and providing immunological protection.^1–3^ However, unlike blood circulatory system diseases, lymphatic system diseases are generally not life-threatening. Consequently, since the initial discovery of the lymphatic system by Gaspare Aselli 400 years ago,^4^ related research progress has been slow.^5^

**Fig. 1:**
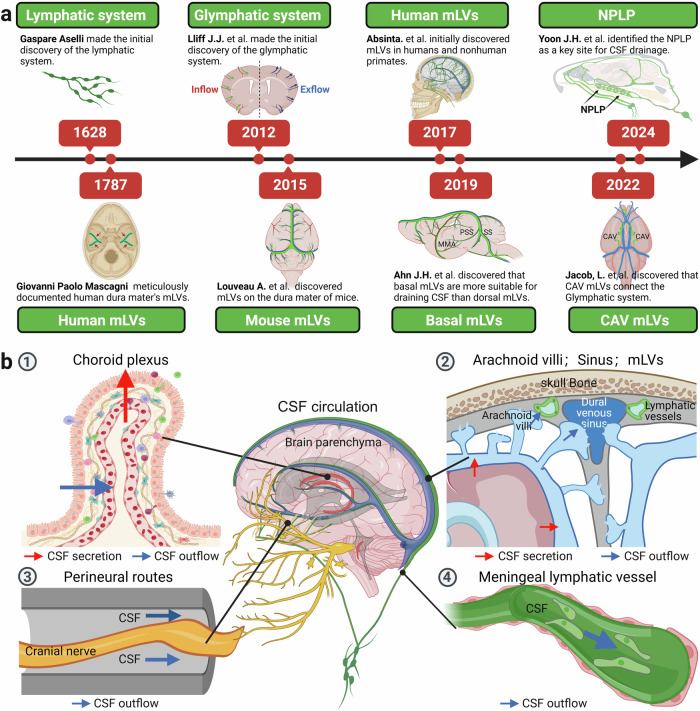
**a** A timeline of key breakthroughs and milestone events in the research history of meningeal lymphatic vessels and meningeal lymphatic drainage (1628: Gaspare Aselli made the initial discovery of the lymphatic system^4^; 1787: Giovanni Paolo Mascagni meticulously documented human dura mater’s mLVs^6^; 2012: Lliff. et al. made the initial discovery of the glymphatic system^14^; 2015: groundbreaking research by Dr. Alitalo’s^16^ and Dr. Kipnis’s teams^17^ independently demonstrated that mLVs are capable of draining CSF and clearing macromolecules. 2017: Absinta et al. initially discovered mLVs in humans and nonhuman primates^86^; 2019: Ahn et al. discovered that basal mLVs are more suitable for draining CSF than dorsal mLVs^78^; 2022: Jacob et al. discovered that CAV mLVs connect the Glymphatic system^80^;2024: Yoon et al. identified the NPLP as a key site for CSF drainage.^82^). **b** CSF circulation. ① Schematic diagram of the structure of the choroid plexus, with red arrows indicating CSF secretion and blue arrows indicating the pathways of CSF absorption. ② Relationships between the brain parenchyma, subarachnoid space, and venous sinuses, with red arrows indicating CSF secretion and blue arrows indicating the pathways of CSF absorption. ③ Perineural space, with blue arrows indicating the pathways of CSF absorption. ④ Schematic diagram of the microstructure of dCLNs, with blue arrows indicating the absorption of CSF. (Created with BioRender.com)

The debate over the existence of a lymphatic system within the central nervous system (CNS) has persisted for more than two centuries. As early as 1787, the Italian anatomist Giovanni Paolo Mascagni provided a detailed description of meningeal lymphatic vessels (mLVs) in the human dura mater.^6^ However, his work was not widely disseminated or recognized within the scientific community, as it was not translated into English. Consequently, prior to 2015, the prevailing scientific consensus was that the CNS lacked a lymphatic system. However, during this period, a group of scholars focused on mLVs.^7–12^ The fluid balance in the CNS is primarily maintained by the cerebrospinal fluid (CSF), which circulates 3–4 times daily, providing support to the brain and removing waste.^13^ Recent studies have revealed that rodents possess a glymphatic system capable of exchanging CSF via aquaporin-4 (AQP4) channels, challenging the traditional theory of CSF circulation.^14^ Further study findings indicated that the CSF produced via this glymphatic system exchange accounts for approximately 20% of total CSF production.^15^ In 2015, groundbreaking research by Dr. Alitalo’s^16^ and Dr. Kipnis’s teams^17^ independently demonstrated that mLVs are capable of draining CSF and clearing macromolecules. Research has also indicated that CSF can exit the skull through the perineural spaces of nearly all cranial nerves.^18^ Furthermore, studies on intraventricular hemorrhage (IVH) model mice suggest that the overexpression of Na–K–Cl cotransporter 1 in the choroid plexus can increase the absorption of CSF.^19,20^ With these findings, the pathways of CSF circulation are becoming clearer: CSF produced by the choroid plexus and brain parenchyma is drained through AGs,^21^ perineural spaces of cranial nerves,^21,22^ mLVs,^21^ and pathways involving the choroid plexus itself^19,20^ (Fig. 1b). Previous studies have indicated that AGs are the primary pathway for CSF drainage, but currently, the paradigm has been altered. Early animal experimental findings have indicated that approximately 50% of the total CSF volume is drained through deep cervical lymph nodes (dCLNs).^21,23,24^ Therefore, modulating the drainage of mLVs may be an effective strategy for treating neurological diseases.

To date, research has elucidated the extensive and multifaceted roles of mLVs and is advancing swiftly (Fig. 1a). Recent research has suggested that meningeal lymphatic drainage dysfunction is involved in the pathogenesis of various neurological diseases, including neurodegenerative diseases,^23,25–40^ traumatic brain injury (TBI),^41–45^ stroke,^46–56^ infections,^57–62^ tumors,^63–67^ functional neurological disorders^68,69^ and hydrocephalus.^46^ However, the current understanding of the regulatory and injury mechanisms of meningeal lymphatics under physiological and pathological conditions is limited. Given the importance of a profound understanding of the interactions between meningeal lymphatic drainage and CNS diseases, this review covers seven key aspects: the development and structure of mLVs, methods for observing mLVs, the function of meningeal lymphatics, the molecular mechanisms underlying meningeal lymphatic injury, the relationships between meningeal lymphatics and CNS diseases, potential regulatory mechanisms of meningeal lymphatics and conclusion and outstanding questions. The aim of this review is to provide new directions for future research and therapeutic strategies related to meningeal lymphatics.

## Development, structure, and distribution of mLVs

Intracranial mLVs resemble systemic lymphatics in their composition of permeable capillaries and larger collecting vessels but are distinct in their single-layered, loosely connected endothelial structure without smooth muscle or a continuous basement membrane, which is crucial for tissue fluid regulation and immune cell migration. In 2007, Baluk et al. reported that endothelial junctions in initial lymphatics are oak leaf shaped with button-like connections, unlike the continuous zipper-like junctions in collecting vessels, allowing fluid entry and leukocyte migration without compromising junction integrity, all of which involve tight junction proteins such as claudins and VE-cadherin.^70^ Since the groundbreaking study of mLVs was published by Dr. Alitalo’s group^16^ and Dr. Kipnis’s group^17^ in 2015, scholars have widely held that mLVs are located primarily adjacent to the venous sinuses of the dura mater; however, recent research has suggested that mLVs may have a more extensive distribution.

### Development of mLVs

The specific markers of lymphatic endothelial cells (LECs) include Lyve-1, Prox1, PDPN, VEGFR3, and CCL21.^71–73^ The development of lymphatic vessels has long been a controversial topic. Studies from the 1900s indicated that the initial lymphatic vessels originate from venous endothelial cells, which then proliferate into adjacent tissues and organs. Another view posits that lymphatic vessels arise from undifferentiated mesenchymal cells, subsequently forming connections between the jugular veins. In 2007, Srinivasan et al. used Prox1-CreERT2 mouse embryos for genetic lineage tracing to determine that LECs originate from the lymph sacs of cardinal veins and proliferate and migrate to form the entire lymphatic system.^74^ Research by Yamaguchi et al. in 2024 revealed that human LECs mainly differentiate from the venous endothelium through the expression of the transcription factor Prox1,^71^ providing high-level evidence for the embryonic tissue origin of LECs. Since then, the notion that peripheral LECs originate from the venous endothelium has been widely accepted by scholars. The origin of intracranial mLVs has long been unclear. Recent studies by Antila et al. in mice have shown that mLVs in mice first appear postnatally around the foramen magnum and intervertebral foramina and grow along blood vessels, cranial nerves, and spinal nerves to various parts of the meninges surrounding the CNS.^75^ Currently definitive evidence that mLVs develop from peripheral lymphatic vessels is unavailable. However, unlike peripheral lymphatic vessels which develop during the embryonic stage, mLVs develop postnatally. VEGF-C and VEGFR3 are crucial for the development of mLVs, but the absence of VEGF-D does not impact their formation. In adult mice, mLVs undergo regression following the depletion of VEGF-C or VEGFR3, administration of the tyrosine kinase inhibitor sunitinib, or the expression of a VEGF-C/D trap, which also impairs lymphatic drainage function. Conversely, supplementation with VEGF-C induces meningeal lymphangiogenesis.^75^ The VEGF-C-VEGFR3 signaling axis is pivotal for the development of mLVs, thus representing a potential target for modulating the plasticity of mLVs or enhancing their drainage capabilities.

### Structural distribution of mLVs

**Fig. 2:**
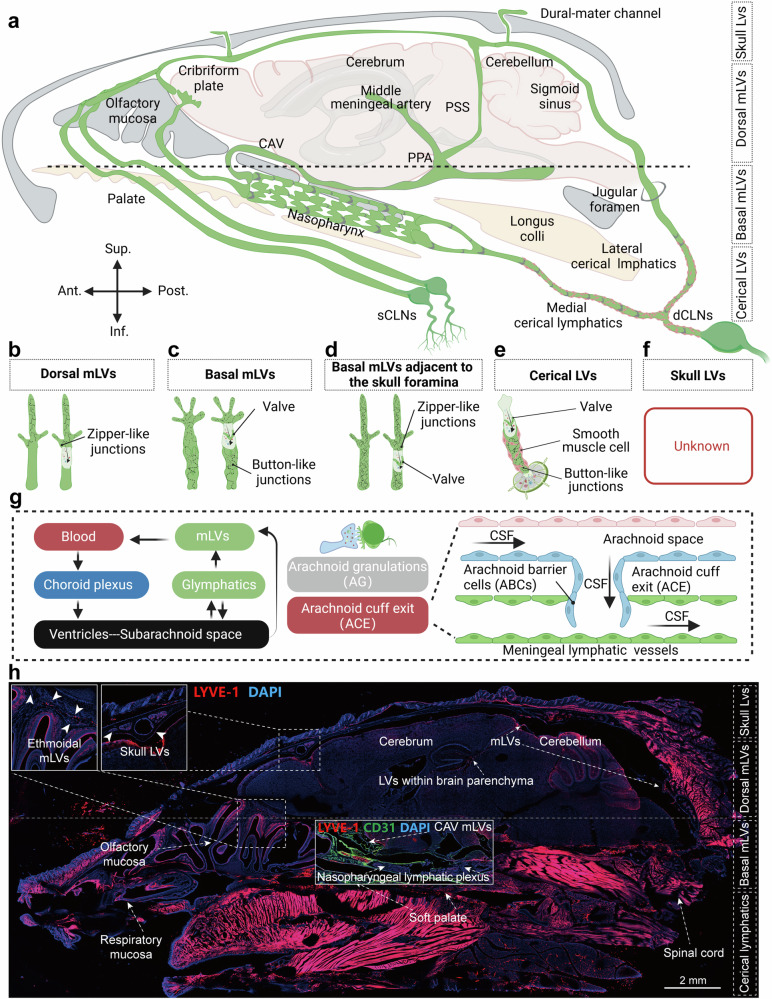
Overview of mLVs in mice. **a** Schematic illustration of the distribution of mouse mLVs. **b** Characteristics of dorsal mLVs (lacks smooth muscle wrapping, devoid of lymphatic valves, and has zipper-like junctions). **c** Characteristics of basal mLVs (lacks smooth muscle wrapping, possesses lymphatic valves, and has button-like junctions). **d** Characteristics of basal mLVs adjacent to the foramina of the skull(increased prevalence of zipper-like junctions and are interspersed with valves akin to those in peripheral organ collecting lymphatics). **e** Characteristics of cerical LVs (enveloped by smooth muscle, equipped with lymphatic valves, and featuring button-like junctions). **f** Characteristics of skull LVs. **g** Schematic illustration of CSF circulation and the structures of AGs and ACE. **h** Representative image of whole-mouse-brain sagittal LYVE-1 and CD31 immunofluorescence staining. Image courtesy of the author’s research group (Not published anywhere). (**a**–**g** Created with BioRender.com)

mLVs share similarities with peripheral lymphatic vessels but also present unique features. Previous studies have indicated that the function of lymphatic vessels is associated with the organ and location in which they are located.^3,70,73,76,77^ Given the absence of literature on the classification of mLVs, the authors propose a categorization of meningeal lymphatic system mLVs on the basis of their function and location as follows (Fig. 2a–f and Table 1):

**Table 1:** Location, classification, and structural characteristics of the meningeal lymphatic system

Classification/Location	Intracranial/Extracranial	Lymphatic valve	SMC	Endothelial cell junctions	Reference
Dorsal mLVs	Intracranial	NO	NO	Zipper-like junctions	^78^
Basal mLVs	Intracranial	Yes	NO	Button-like junctions	^78^
Basal mLVs adjacent to skull foramina	Intracranial	Yes	NO	Zipper-like junctions	^78^
Ethmoidal mLVs		–	–	–	^80^
Nasopharyngeal lymphatic plexus	Extracranial	Yes	NO	Button-like junctions	^82^
Medial cervical lymphatics	Extracranial	Yes	Yes	Button-like junctions	^82^
Lateral cervical lymphatics	Extracranial	Yes	Yes	Button-like junctions	^82^

#### Dorsal mLVs

According to a study published in 2019 by Ahn et al., mLVs on the dorsal aspect of the skull mostly exhibit a continuous zipper-like connection pattern and are morphologically immature. Using Prox1-GFP mice, a research group revealed that the mLVs located adjacent to the sagittal and transverse sinuses on the dorsal aspect of the skull have smaller diameters and that their tubular structures are largely discontinuous. Moreover, the majority of dorsal skull mLVs are clustered within the dural folds enveloping the sagittal and transverse sinuses without being stretched, and they lack lymphatic valves and smooth muscle cells (Fig. 2b).^78^ Research on tumor-bearing mouse models indicates that disruption of mLVs located dorsal to the skull impairs CSF drainage and the migration of tumor cells to dCLNs, indicating their critical role in tumor-associated immune processes.^64^ This findings suggests that the structural integrity of dorsal mLVs not only facilitates entry but also may participate in extensive intracranial immune regulation. However, it remains unclear whether the functional characteristics of dorsal mLVs are associated with their zipper-like junctions and morphological immaturity. In addition to the absorption of CSF by dorsal mLVs, recent research has highlighted the arachnoid cuff exit (ACE) as a pivotal structure for fluid and molecule exchange between the CNS and dural compartments, which is crucial for neuroimmune communication and CSF drainage.^79^ Overall, dorsal mLVs participate in the drainage of CSF and extensive immune regulation (with a structure potentially more conducive to the entry of immune cells). However, the proportion of CSF absorbed by dorsal mLVs is currently unclear likewise, the proportion of CSF drainage mediated by the ACE requires further investigation.

#### Basal mLVs

**Fig. 3:**
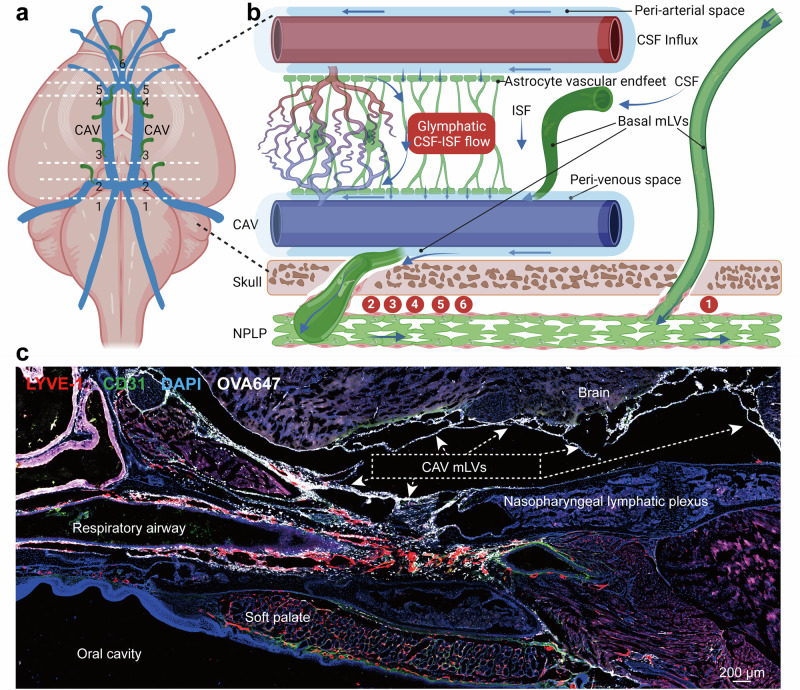
Cavernous lymphatics. **a** Schematic of the CSF drainage pathways of glymphatic-mLVs in the mouse CAV. The cerebral veins and dural sinuses (depicted in blue) serve as conduits for blood drainage from the brain. **b** Sites 1–6 represent newly identified mLV uptake locations where the perivenous efflux of glymphatic fluid from these regions communicates with the mLVs. (**a**, **b** Created with BioRender.com.). **c** Representative fluorescence staining images of LYVE-1 and CD31 in the mouse CAV, area 1 hour after intracisternal injection of OVA647, courtesy of the author’s research team (Not published anywhere)

In mice, the mLVs located adjacent to the petrosal sinus and sigmoid sinus at the base of the skull exhibit larger diameters and an abundance of protruding capillary lymphatic branches (primary lymphatic vessels). These capillary lymphatics have blind ends with characteristic oak leaf-shaped, button-like junctions and are equipped with lymphatic valves but are devoid of smooth muscle coverage (Fig. 2c).^78^ The basal skull mLVs, characterized by button-like junctions and the profusion of primary lymphatic branches, are structurally optimized for CSF drainage, a function that deteriorates with age owing to endothelial damage. Given the complexity of skull base architecture, the connections and functional characteristics related to lymphatic drainage and CSF outflow in other disease models are not yet well understood.^78^ In 2022, Jacob et al. discovered an expanded network of anterior mLVs surrounding the cavernous sinus (CAV) at the base of the mouse skull.^80^ This network of lymphatic drainage around the CAV connects with dorsal and basal lymphatics and is drained through the foramina and fissures of the skull. Cavernous lymphatics specifically drain perivenous effluxes from their tributary cerebral veins into superficial cervical lymph nodes (sCLNs) and dCLNs, thereby providing region-specific drainage of the glymphatic outflow from the dura mater to the cervical LNs.^80^ In the posterior region of the CAV, three lymphatic foci (Sites 1–3) were identified, and an additional three mLV foci (Sites 4–6) were observed in the rostral part of the CAV, as shown in Fig. 3a–c.

Adjacent to the foramina of the skull, basal mLVs feature an increased prevalence of zipper-like junctions and are interspersed with valves akin to those in peripheral organ-collecting lymphatics. However, these mLVs exhibit a mixed phenotype indicative of both capillary and collecting lymphatic vessels, with variability in their junctional patterns and LYVE-1 expression levels. Additionally, a defining characteristic of these mLVs is the absence of a surrounding smooth muscle cell layer (Fig. 2d).^78^ Overall, basal mLVs are larger in diameter, are interconnected via button-like junctions, and are also equipped with lymphatic valves. Studies indicate that basal mLVs serve as pivotal conduits for CSF drainage; however, the reasons why basal mLVs are central to CSF outflow remain unclear. It is uncertain whether this is related to their structural characteristics. Another unresolved issue pertains to the specific roles that basal mLVs, which are adjacent to the foramina of the skull, play in CSF drainage and immunological functions.

#### Ethmoidal mLVs

The cribriform plate represents a significant pathway for CSF outflow within the mouse skull.^81^ However, the role of mLVs in mediating this drainage through the cribriform plate remains to be conclusively established.^21^ Intriguingly, ethmoidal mLVs form dorsal connections with rostral projections of superior olfactory sinus mLVs and ventral connections with CAV mLVs. Ethmoid mLVs display no association with the dural sinuses and do not traverse the ventral or central portions of the cribriform plate toward the nasal cavity. Given these anatomical considerations, ethmoidal mLVs might play a modest role in mediating nasal CSF outflow via the cribriform plate (Fig. 2h).^80^

#### Nasopharyngeal lymphatic plexus *(NPLP)*

In 2022, Decker et al. utilized magnetic resonance imaging (MRI) to show that the NPLP is a major conduit for CSF drainage in mice, and the findings also indicated a reduction in drainage to dCLNs with aging.^26^ Yoon et al. in 2024 discovered a unique NPLP in the mucosa of mice and macaques (nonhuman primates), and this NPLP is actually a network of lymphatic vessels located in the nasopharynx that generally resembles an inverted saddle. This structure is similar to that of peripheral primary lymphatic vessels, consisting of short lymphatic vessels with valves but lacking smooth muscle cell encapsulation (Fig. 2a, h).^82^ To elucidate the drainage pathways further, the research team administered multiple tracers, such as TMR-dextran, were injected into the cisterna magna of PROX1-GFP mice, and the deposition of these tracers in lymphatic vessels and nodes was observed. The results indicated that the NPLP drains CSF downward through the medial and lateral deep cervical lymphatic vessels to the dCLNs. Notably, the medial deep cervical lymphatics emerged as the predominant drainage route, carrying a flow volume 180% greater than that of the lateral deep cervical lymphatics.^82^ Hence, the NPLP serves as a major hub for the drainage of CSF from mLVs to dCLNs. The NPLP collects CSF draining from the subarachnoid space out of the skull primarily from three sources: the pituitary region and CAV area of the middle cranial fossa, as well as other regional lymphatic vessels (including the cribriform plate and anterior cranial fossa). Tracers flowing to scLNs do not pass through the NPLP. This array of findings not only emphasizes the critical role of the NPLP in CSF drainage but also highlights the intricate structural and functional landscape of the lymphatic system, particularly in relation to aging processes. An intriguing question that remains is the precise proportion of CSF drainage attributed to the NPLP, which is currently unclear. Additionally, the role of the NPLP in immunological functions warrants further investigation. Furthermore, it is critical to address the ongoing debate surrounding whether the NPLP constitutes a component of mLVs.

#### Dural lymphatics distant from the venous sinuses

**Fig. 4:**
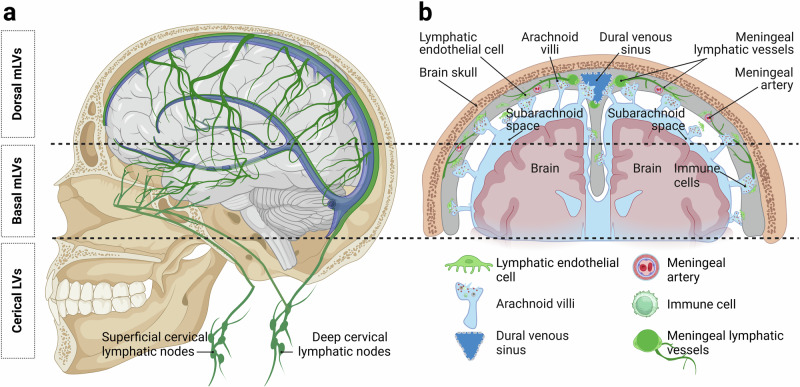
Distribution and characteristics of human mLVs. **a** Representation of the comprehensive distribution of the meningeal lymphatic system. **b** Lymphatic endothelial cells are extensively present within the dura mater, and a close relationship is observed between AGs and LECs. (a, b Created with BioRender.com)

Despite the classical theory that mLVs predominantly reside adjacent to dural venous sinuses since their discovery, emergent research suggests a more extensive distribution throughout the dura mater. Specifically, recent research by Vera Quesada et al. indicated that mLVs in the human dura mater are more extensively distributed in areas of the dura mater that are distant from the venous sinuses (Fig. 4a, b).^83^ Building upon these observations, the research team has identified three distinct categories of mLVs within the human dura mater: (i) lymphatic vessels closely associated with blood vessels; (ii) lymphatic vessels that are not near blood vessels; and (iii) aggregates of LYVE-1-positive cells that are distributed among blood vessels (Fig. 4b),^84^ thus advancing our understanding of dural lymphatic complexity. Nonetheless, whether these three types of lymphatic vessel expression represent different structural forms of mLVs and whether they perform discrete functions remain unclear at present. Further research is needed to elucidate the specific roles and characteristics of each LYVE-1-positive mLV category within the dural microenvironment.

#### Relationships between AGs and mLVs

The traditional view holds that AGs, also known as arachnoid villi, regulate the drainage of CSF by passively transporting CSF to the dural venous sinuses. In 2015, Louveau et al. reported the presence of mLVs adjacent to venous sinuses.^17^ However, the relationship between AGs and mLVs has remained unclear. Previous studies have shown that AGs are connected to the body through a basal stalk, with the body consisting of a core, a capsule, and one or more apical dome regions.^85^ In 2017, Absinta et al. documented the presence of mLVs near the dural venous spaces through systemic injection of the tracer gadobutrol (approximately 600 kDa).^86^ In 2023, research by Shah et al. on the microstructure of AGs in the human brain revealed that the peripheral regions of AGs were positive for collagen, arachnoid, macrophages, mast cells, lymphocytes, plasma cells, vascular endothelium, and rare synaptic markers. The core region of AGs contains collagen, arachnoid, macrophages, mast cells, LECs, plasma cells, vascular endothelium, and rare synaptic marker components.^87^These findings suggest the direct transport of CSF from the subarachnoid space to the meningeal lymphatic system via AGs (Fig. 2g).

### Extracranial lymphatics associated with mLV drainage

#### Cervical lymphatics

The cervical lymphatics, a crucial component in the drainage of CSF, comprise both medial and lateral cervical lymphatics. Initially, the medial cervical lymphatics are endowed with semilunar valves spaced at intervals ranging from 250 to 750 µm, which are directed toward the LNs. These vessels are lined with a thick, albeit patchy, layer of circular cells expressing α-smooth muscle actin and are responsive to modulation through the nitric oxide(NO) pathway and α-adrenergic stimulation.^82^ Concurrently, the lateral cervical lymphatic vessels (lateral dCLVs), which originate from the basolateral dura and extend through the jugular foramen to the dCLNs, feature semilunar valves and smooth muscle coverage similar to that of medial cervical lymphatics, and lateral dCLVs are equipped with contractile lymphangions capable of propelling lymph toward LNs via spontaneous cyclical contractions,^88^ serving as pathways for CSF drainage to dCLNs (Fig. 2a, e).^82^ Research suggests that the medial deep cervical lymphatics function as the main drainage pathway, with a flow volume that is 180% greater than that of the lateral pathways.^82^ It is currently unclear whether the difference in drainage volume between these regions is due to their distinct upstream connections. The presence of smooth muscle cells on the surface of the cervical lymphatics enables the potential modulation of intracranial CSF drainage by regulating the contraction and relaxation of these smooth muscles, thus presenting a potential therapeutic target in pathological conditions.

#### Ocular lymphatic system

The ocular lymphatic system, particularly within the posterior chamber of the eye, has recently drawn significant attention for its role in linking central and peripheral immunity. In 2024, Yin et al. discovered that a compartmentalized lymphatic system within the eye can mediate eye–brain immunity. Distinct lymphatic drainage systems exist in the anterior and posterior parts of the eye, with the latter being connected to the mLVs through the lymphatic system within the optic nerve sheath, sharing a lymphatic circuit and establishing a unified immune response between the posterior eye and the brain.^89^ During brain infection with herpes simplex virus, vitreal immunity in the eye can protect the mouse brain from viral assault, and this protective effect is not limited to viral infections; bacterial infections and tumors can also activate vitreal immunity to protect the brain.^89^ In summary the lymphatic system of the posterior chamber of the eye joins the meningeal lymphatic network of the CNS at the dCLN. The optic nerve sheath lymphatic system network can drain antigens inoculated into the vitreous body to the dCLN and initiate a local protective immune response in the brain. Therefore, the ocular lymphatic system is not only a potential therapeutic target for ocular diseases^90^ but may also represent a promising candidate for therapeutic strategies targeting other CNS disorders. With the advancement of research on the ocular lymphatic system, it will become one of the hotspots in future studies on the interaction between central and peripheral immunity.

#### Dura-mater channels

**Fig. 5:**
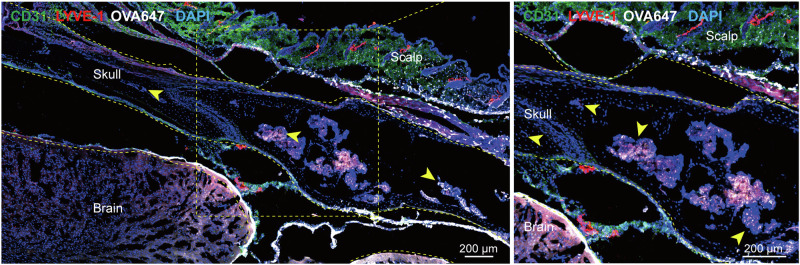
Mouse skull lymphatics. Image of immunofluorescence staining LYVE-1 and CD31 on the posterior aspect of the mouse sagittal sinus following the injection of OVA647 into the cisterna magna; the image provided by the author’s research group and has not been published anywhere. (yellow arrows indicate instances where skull LVs and OVA647 are co-labeled)

The intricate communication between the CNS and the bone marrow is highlighted by a recent study from Mazzitelli et al., which underscores the pivotal role of CSF in this interplay. This study revealed that CSF permeates through dural channels into the cranial bone marrow, where it influences a variety of cells within the bone marrow microenvironment. In the context of disease-related injury, CSF-derived signals increase the generation of cells in the bone marrow and their dissemination into the meningeal spaces.^91^ This finding elucidates a CSF-based mechanism of communication between the CNS and bone marrow, which plays a role in regulating CNS immune responses.^91^ Moreover, the authors’ research team discovered LYVE-1-positive cells within the skull for the first time that were predominantly located in the bone marrow cavities (Fig. 2f, h). Further investigation by injecting OVA647 into the cisterna magna revealed an association between LYVE-1-positive cells within the skull and OVA647 fluorescence, and the deposition of OVA647 was also observed in the scalp lateral to the sagittal sinus outside the skull (Fig. 5). This is particularly compelling, considering that existing human imaging studies have already shown that intracranial lymphatic fluid can directly connect with the scalp’s lymphatic vessels through the emissary veins of the skull.^80^ Hence, whether the dura mater channels adjacent to the sagittal sinus are directly connected to extracranial lymphatic vessels through mLVs remains an essential topic for further exploration, potentially opening new frontiers in our understanding of CNS immune regulation and CSF drainage.

## Methods for observing the meningeal lymphatic system

A deep understanding of mLVs is highly important for the diagnosis and treatment of CNS diseases. However, current clinical methods for observing mLVs, as well as laboratory animal research methodologies, remain limited. This section presents the methodological advances in the study of mLVs in both humans and animals.

### Methods for observing human lymphatic vessels

Because lymphatic diseases are not typically fatal, visualizing the lymphatic network is challenging, and its unidirectional flow hinders the use of straightforward visualization and techniques such as the use of injectable dyes; therefore, lymphatic imaging remains less advanced than vascular imaging. Lymphangiography necessitates the design of tracer agents for interstitial injection that are readily taken up by the lymphatic vessels.^92–94^

#### Methodologies for the clinical assessment of peripheral lymphatic vessels

In recent years, peripheral lymphatic imaging technologies have rapidly evolved. Currently, the lymphatic imaging techniques employed in clinical practice include X-ray lymphography,^95–98^ lymphoscintigraphy,^99–101^ SPECT/CT,^102–105^ near-infrared (NIR) lymphography,^106–109^ magnetic resonance (MR) lymphography,^110,111^ and photoacoustic imaging.^112^ The advantage of X-ray lymphography lies in the effective tissue penetration of X-ray waves; however, its invasiveness^113–115^ and procedural complexity render it less commonly used in peripheral clinical settings. Lymphoscintigraphy serves as the standard method in clinical lymphatic imaging and is extensively utilized for sentinel lymph node localization^116,117^ following the interstitial injection of radioactive tracers for several cancers. Although it provides only two-dimensional images and is unable to precisely delineate anomalies of the lymphatic system and the position of the LNs, this limitation can be overcome by combining this method with SPECT/CT imaging technology.^118,119^ However, such technology requires expensive and bulky equipment and is thus not readily applicable for short-term human mLV observation. NIR lymphography is a relatively newer technique that was first used in humans approximately 19 years ago,^108,109^ and its noninvasive nature makes it a potential approach for future observation of cranial mLVs. Magnetic resonance lymphangiography (MRL) is a convenient and safe technique that delivers comprehensive information on lymphatic vessels and veins, aiding in the planning of treatment for lymphaticovenous anastomosis (LVA) and the assessment of lymphedema complications,^120^ and it has already been applied to observe mLVs. Photoacoustic imaging is a promising technique that can utilize safe and mature optical tracers (such as ICG) for real-time imaging of lymphatic structures, boasting an imaging depth of up to 2.5 cm and a fine spatial resolution of approximately 160 micrometres. Recent reports have documented the application of photoacoustic imaging in observing peripheral lymphatics,^112,121^ indicating its potential for future observation of human mLVs.

In summary, MRI is typically the preferred method for observing human peripheral and meningeal lymphatics.^122^ Although NIR technology and stereoscopic wide-field photoacoustic microscopy have been utilized to visualize peripheral lymphatics and recent studies have demonstrated that these technologies^123,124^ can be used to study and observe the dynamic drainage of mLVs in mice, there is a lack of documentation on the application of these technologies for observing human mLVs. Borth NIR and stereoscopic wide-field photoacoustic microscopy are potential noninvasive methods for observing human mLVs. Future research is needed to validate the efficacy and safety of NIR and stereoscopic wide-field photoacoustic microscopy in human studies and explore their potential in enhancing our understanding of the anatomy and function of human mLVs. Addressing these gaps could unlock new avenues for diagnosing and treating conditions related to lymphatic dysfunction in the human brain.

#### Noninvasive methods for observing the human meningeal lymphatic system

Recent advances in noninvasive imaging for visualizing human mLVs are spearheading neuroscience research, despite challenges posed by their minuscule structure. Key techniques include contrast-enhanced MRI and contrast agent-free MRI to study mLVs.

Recent studies have shown that the use of gadobutrol-enhanced MRI following an intravenous injection of gadobutrol is an effective method for observing mLVs. Gadobutrol, a gadolinium-based contrast agent, is highly permeable and can penetrate the permeable endothelial barrier of lymphatic vessels. On cranial and spinal MR images, tissue enhancement effects are typically visible within approximately 15 min of gadobutrol administration and can generally persist up to 45 min postinjection. In 2017, Absinta et al. employed T1-weighted black-blood and 3D T2 fluid-attenuated inversion recovery (T2-FLAIR) imaging after an intravenous injection of gadobutrol, marking the first observation of mLVs in both humans and primates.^86^ In 2021, Wu et al. used 0.2 mmol/kg gadobutrol-enhanced T2-FLAIR imaging to visualize mLVs in patients with reversible cerebral vasoconstriction syndrome (RCVS) in remission and those with cluster headaches without comparing mLV drainage volumes between the groups.^125^ In the same year, Ding et al., utilizing gadobutrol-enhanced MRI and intravenous administration of gadobutrol, reported that patients with idiopathic Parkinson’s disease (PD) exhibited significantly diminished flow through the mLVs along the superior sagittal and sigmoid sinuses, along with a marked delay in perfusion to the dCLNs, compared with patients with atypical parkinsonism.^37^ In their 2022 study, Jacob et al. used real-time vessel wall MRI to evaluate neurological patients after intravenous gadobutrol injection and reported an elaborate anterior mLV network encircling the CAV connected to the dorsal and basal lymphatic routes, with exit points via the foramina of the emissary veins.^80^ These findings underscore the indispensable role of mLVs in glymphatic system uptake and outflow processes from perisinusal and perivenous areas.^80^ Research findings further indicated a significant variance in the mLV between sexes, whereas no such variance was observed across patients with different neurological disorders.^80^ In their 2023 study, Albayram et al. proposed that interstitial fluid drainage is detectable on routine MRI, with interstitial fluid routing from the brain parenchyma through cortical perivenous spaces to the dural meningeal lymphatics along the superior sagittal sinus (SSS) in a trajectory that is separate from CSF circulation. Their research additionally revealed that the key locations for glymphatic clearance to meningeal lymphatics in humans are chiefly found along the SSS, with an emphasis on the posterior area.^126^ The findings of the 2023 study by Sennfält et al. indicated that dynamic intravenous contrast-enhanced MRI can be used to visually assess the compromised drainage function of the glymphatic‒meningeal lymphatic system in patients with cerebral small vessel disease.^127,128^ In 2023, Wang et al. conducted a study on patients with intracranial tumors via dynamic contrast-enhanced T1 black blood sequences and reported that long-term impairment of mLV drainage function is a risk factor for tumor progression.^67^ Zhang et al.’s 2024 study correlated mLV drainage dysfunction with increased cranial subdural hematoma (CSDH) recurrence, as evidenced by MRI.^50^ The aforementioned studies indicate that gadobutrol-enhanced MRI is a reliable method for observing mLVs, and the utilization of this technique has greatly advanced the field of mLV research.

In light of the potential side effects associated with the injection of contrast agents, scientists have recently been investigating new noninvasive MRI techniques for the observation of mLVs. In 2022, Albayram et al. utilized 3D T2-FLAIR MRI, leveraging endogenous signals from protein-rich lymphatic fluid rather than exogenous contrast agents, to map human dural lymphatic structures and revealed direct links between lymphatic channels alongside cranial nerves and vascular structures to CLNs, as well as age-related CLN atrophy and thickening of lymphatic channels in the dorsal and ventral regions.^129^

In summary, noninvasive methods for observing the flow velocity of mLVs are reliable. Although there are potential risks associated with the injection of contrast agents, the majority of studies observing human mLVs currently employ the use of gadobutrol-enhanced MRI (Table 2). In contrast, MRI methods that do not involve the injection of contrast agents for the observation of mLVs require further investigation. At present, it is unclear whether there are differences in the outcomes observed by the two methods for mLVs, and a deeper exploration into their respective advantages and limitations is warranted.

**Table 2:** Noninvasive investigations of the human meningeal lymphatic system

Year	The type of study	Number of patients	Number of controls	Enhancer	MRI observation sequence	Apparatus	Time point of observation	Injection dose	Reference
2017	–	–	5 (3 females)	Gadobutrol	2D T1-weighted black blood; contrast-enhanced 3D-FLAIR	3 T MRI	31 min after the intravenous injection	0.1 mol/kg	^86^
2021	Prospective study	35 individuals with cluster headache gadolinium	182 RCVS	Gadobutrol	CE-T2-FLAIR	3 T MRI	following a continuous 18-hour period	0.2 mmol/kg	^125^
2021	Prospective study	375 patients with idiopathic Parkinson’s disease (iPD)	19 patients with atypical Parkinsonian (AP)	Gadobutrol	2D T1-weighted black-blood; 3D T1 weighted black blood; contrast-enhanced 3D-FLAIR	3 T MRI	30 min	0.1 mol/kg	^37^
2022	Retrospective study	11 patients (5 with idiopathic intracranial hypertension ; 4 with multiple sclerosis; 1 with unilateral jugular stenosis;1 with Gorham–Stout disease)	0	Gadobutrol	real-time vessel-wall magnetic resonance imaging ; contrast-enhanced 3D-FLAIR	3 T MRI	≥15 min	0.1 mol/kg	^80^
2023	Prospective study	20 patients with arteriosclerotic small vessel disease	15	Gadobutrol	3D T1-weighted MRI contrast-enhanced 3D-FLAIR	3 T MRI	0 h, 1 h, 4 h, 8 h, 24 h)	0.175 mol/kg	^127^
2023	Retrospective study	19 patients with small vessel disease	0	Gadobutrol	2 D coronal T2-FLAIR	3 T MRI	20-30 min	0.1 mol/kg	^126^
2023	Prospective study	146 patients with intracranial tumor patients	100 individuals without intracranial tumor	Gadobutrol	T1 Black blood	3 T MRI	3 h	0.1 mol/kg	^67^
2022	Retrospective study	81 (epilepsy patients or those with a suspicion of epilepsy)	0	–	3D FLAIR	3 T MRI	–	–	^129^
2023	Prospective study	27 patients with CSDH	7	–	3D T2-FLAIR	3 T MRI		–	^50^

### Methods for studying mouse mLVs

With advancements in the study of the structure and function of mLVs, methods for examining mLVs in animal models have become a current research focus. Techniques for observing mLVs in mice can be categorized into in vivo and ex vivo approaches. The in vivo methods for observing mLVs in mice include two-photon microscopy, transcranial microscope imaging, and photoacoustic microscopy in living tissue preparations. Ex vivo methodologies for mLV observation include brain section microscopy, 3D imaging of solvent-cleared organs (3DISCO), tissue section staining, and electron microscopy.

#### In vivo observation methods for mouse mLVs

##### Two-photon microscopy imaging

Two-photon microscopy is widely used to observe peripheral lymphatic systems.^130^ In 2013, Xie et al. utilized two-photon technology to observe the exchange between CSF and interstitial fluid.^131^ In vivo two-photon microscopy equipped with various probes can monitor CSV tracers at the microscale.^131,132^ Although two-photon imaging can be used to observe the drainage of CSF toward CLNs,^23,78^ it currently cannot be used to differentiate mLVs from the resulting drainage fluid directly, requiring the combination with Prox1-GFP transgenic mice to observe mLVs and CSF drainage.^17^ Since two-photon imaging typically involves a craniotomy, the surgery may damage dura mater or brain tissue. Furthermore, the current technology provides tracers with limited visibility within the brain and has shortcomings such as low image resolution.

##### Transcranial microscope imaging

NIR light sheet microscopy within the NIR-II spectrum (1000–1700 nm) represents a novel imaging modality extensively applied to observe deep tissues in small animals, including brain tissues,^133,134^ and is utilized to visualize immune cells within LNs. This technique allows for deep tissue penetration and yields images with high resolution. NIR-II nanoprobes enable dynamic observations of the regulation of CSF inflow and CSF drainage through submandibular LNs into the periphery.^135^ In 2021, Cardinell et al. used NIR techniques and novel contrast agents to study the drainage of tracers from the eyes to the neck, validating their presence in CLNs through postmortem fluorescence imaging.^136^ In 2024, Sun et al. deployed NIR-II nanoprobes to investigate the functionality of the glymphatic system in mice under anesthesia and cerebral ischemia‒reperfusion injury conditions and reported that the functionality of the glymphatic system was compromised following cerebral ischemia‒reperfusion injury, as evidenced by impaired glymphatic inflow and reduced glymphatic outflow.^123^ In the same year, Li et al. performed NIR-II imaging studies and reported that hypothermia regulates neuroinflammation following brain injury by increasing glymphatic system influx.^137^ The current technology allows imaging of the entire lymphatic system in the brain.^138–140^ Compared with mLV observations via two-photon imaging, NIR-II fluorescence imaging can be used to observe cranial glymphatic systems dynamically, offering the advantages of being noninvasive and providing high-resolution images. Nevertheless, this imaging technology cannot perform real-time imaging of CSF tracer circulation throughout the body.^141,142^ Additionally, direct observation of mLVs is not yet possible with this technology alone, necessitating the combined use of Prox1 genetic tools in mice for mLV investigations. The author suggested that with the emergence of novel nanoprobes, further advancements in the application of NIR imaging technology in mLV research will occur.

##### Photoacoustic microscopy

As a hybrid imaging technique, photoacoustic imaging combines the advantages of optical resolution with acoustic penetration depth and has made progress in brain imaging and glymphatic imaging in recent years. This technique allows imaging of the vasculature and lymphatics of patients’ limbs,^143–146^ aiding in preoperative planning and playing a significant role in the preoperative assessment of the lymphatic vasculature in patients with conditions such as limb edema or aging.^146–148^ Initially, this technique was limited to glymphatic system research because of its inability to distinguish CSF from mLVs.^149^ However, by 2024 Yang et al. visualized the dynamic drainage of mLVs with a stereoscopic wide-field photoacoustic microscope, which features a depth imaging capability of 3.75 mm, identified the peak drainage phase occurring approximately 20–40 min postinjection, and determined the flow direction from the CSF to the LNs. One study reported a 70% reduction in mLV drainage in Alzheimer’s disease (AD) model mice.^124^ In 2020, Suzuki et al. compared photoacoustic lymphangiography and NIR fluorescence cholangiography and reported that photoacoustic imaging, in contrast with NIR fluorescence imaging, provides three-dimensional imaging of lymphatic vessels and has significant advantages.^145^ Future advances in technology will propel research progress in the field of mLVs.

In conclusion, the invasiveness and low image resolution of two-photon imaging have promoted a preference for NIR imaging and photoacoustic imaging techniques are currently the preferred methods for noninvasive observation of mLVs in mice. Owing to current limitations in equipment, MRI techniques are not yet viable options for observing mLVs in mice. Although recent reports have identified the drainage of CSF to CLNs in mice using gadobutrol,^39,78^ distinguishing mLVs from the results is not yet possible. It is therefore essential that future research focus on advancing MRI technology to overcome the present barriers, with the expectation that MRI could ultimately become a valuable tool for the detailed study of mLVs in mice. Continued exploration in this area is crucial to address the unresolved challenges and leverage new insights into the intricate functions of mLVs within the lymphatic system.

#### Ex vivo methods for observing mouse mLVs

Methods for observing mLVs in mouse tissue samples include brain section microscopy, 3DISCO, tissue section staining, and electron microscopy.

##### Brain section microscopy

Fluorescence micro-optical sectioning tomography (fMOST) was used to observe the glymphatic system within the mouse brain. In 2022, He et al. infused brains with fluorescent dextran at 30 and 120 min postinjection, labeled the cerebral vascular system with lectin, and then imaged the resin-embedded brain specimens via an fMOST system. This process revealed the overall 3D configurations of the glymphatic system, illustrating the inflow of CSF and the extrusion of fluid in the brain.^150^ This technique can also be combined with genetically modified mice expressing Prox1-fluorescent reporters to observe mLVs, as well as alterations via the use of primary antibodies coupled to LYVE-1, which can be administered intracisternally at the cisterna magna to study the structure and drainage of mLVs. However, a limitation of this technique is the inability to observe dynamic changes in mLVs in vivo.

##### 3DISCO

Tissue clearing imaging techniques are applicable for observing various tissues and organs, including the brain, spinal cord, immune organs, and tumors, among others. Solvent-based tissue clearing for 3D organ imaging requires a processing time of 3 h, and imaging can be completed within 45 min; this technique can also be used to observe the glymphatic system.^142,151^ In 2022, Jacob et al. utilized 3DISCO technology to discover an expanded network of mLVs around CAVs at the base of the mouse skull; the discovery of CAV mLVs provided direct evidence of the association between the glymphatic system and mLVs.^80^ This technique further enhances the efficiency of observing mLVs compared with fMOST, and the combination of different tracer agents and Prox1 genetically modified mice allows for better observation of mLV structure. Like the fMOST technique, 3DISCO also cannot dynamically observe changes in mLV drainage.

##### Tissue section staining

As previously mentioned, on the basis of their morphology and function, mLVs can be categorized into diverse subclasses, including intracranial mLVs (dorsal mLVs, basal mLVs, basal mLVs adjacent to the skull foramina, skull LVs, and the NPLP) and cervical LVs (cLVs and LNs). Given the relative accessibility of dorsal mLVs and CLNs, the majority of extant studies have focused primarily on these structures. An array of specific markers, such as Lyve-1, Prox1, PDPN, VEGFR3, and CCL21, facilitate the targeted staining of mLVs, thereby allowing their further examination. Dorsal mLVs, characterized by their smaller diameter and lack of lymphatic valves,^78^ contrast sharply with basal mLVs, which not only exhibit a larger diameter but also a more mature morphology, including lymphatic valve structures. Importantly, the morphology of basal mLVs suggests a greater ability to drain CSF.^78^ The presence of lymphatic valves is critical for proper lymphatic function, as denoted by the specific marker FOXC2. However, diminished expression of FOXC2 has been correlated with the development of edema in mLVs,^46^ confirming the importance of such valves. Considering the complexity of the skull base and the traversal of cranial nerves and blood vessels, obtaining the complete structure of the dura mater poses a challenge. Consequently, current research on mLVs has focused predominantly on dorsal mLVs. The pursuit of a comprehensive understanding of mLVs is thus impeded, leaving several pressing questions unresolved. Among them is how to effectively circumvent anatomical complexities to elucidate the full extent of the functions of mLVs and their contributions to neurophysiological and pathological processes. While contention persists regarding the categorization of the NPLP as part of the mLVs, the architectural features of the NPLP undeniably mirror those of the lymphatic vessels situated at the base of the skull. Like the lymphatic ducts in the neck, the NPLP is also sheathed by smooth muscle cells.^82^ Current research on the NPLP requires decalcification treatment before frozen sections can be observed.^82^

Cervical LVs include cLVs and LNs. sCLNs are commonly used to study the drainage mechanism of mLVs^62^: Since sCLNs in mice are located superficially under the skin on the ventral side of the neck, the drainage of tracers to the LNs via mLVs can be observed via small animal in vivo imaging techniques following tracer injection to assess the extent of mLV damage. dCLNs are often used to study drainage mechanisms through mLVs^46^; dCLNs are located behind the sternocleidomastoid muscle adjacent to the trachea and are white or milky in appearance, typically round or oval in shape, with a deeper anatomical position. Animal studies have shown that dCLNs are responsible for 50% of the drainage of CSF,^21^ and they serve as hubs for central and peripheral immune interactions. dCLNs are currently popular targets for regulating mLVs: on the one hand, ligation of dCLNs can be used to block the drainage of mLVs to the periphery and thereby observe central or peripheral inflammation; on the other hand, treatments for brain tumors or dementia through drug delivery to dCLNs and LVA have achieved preliminary success.^152,153^

##### Electron microscopy

Currently, both scanning electron microscopy (SEM) and transmission electron microscopy (TEM) have been used to observe mLVs. For example, in 2021, Rustenhoven et al. observed mouse mLVs using TEM and discovered that the dural sinuses were adjacent to mLVs with discontinuous junctions between the endothelial cells of mLVs, providing ultrastructural evidence for the exchange of fluid and cells between mLVs and the dural sinuses.^154^ In addition, in 2024, the author’s research team utilized SEM to observe lymphatic thrombi within the mLVs of mice with IVH, revealing the mechanism of lymphatic thromboembolism-mediated drainage impairment in mLVs. This study further revealed that mLVs are involved in the development of brain damage and hydrocephalus post-IVH, suggesting that the regulation of mLVs is a potential therapeutic strategy to ameliorate post-IVH hydrocephalus.^46^ However, despite the progress made, questions pertaining to the precise regulatory mechanisms of mLVs and their interactions with other neurovascular structures remain unresolved.

##### Transgenic mice

Transgenic mice expressing a fluorescent molecule play a critical role in studying the formation and development of the lymphatic and vascular systems, as they allow for the characterization of lymphatic or vascular development via real-time imaging throughout experimental protocols.^155,156^ Transgenic fluorescent mouse models such as Prox1-GFP,^157^ VEGFR3-YFP,^158^ Prox1-tdTomato, Flk1-mCherry,^159^ Flk1-GFP, and Flt1-DsRed have been widely employed in research on the lymphatic or vascular systems. Flt1-DsRed mice have been utilized to investigate potential processes guided by blood and nerve cues.^160^

Prox1-GFP transgenic mice have played a significant role in studies investigating the development^75^ and distribution characteristics^17,78,82^ of mLVs. In 2017, Zhong et al. utilized transgenic mice engineered to express GFP in LECs (Prox1-GFP) and DsRed in blood endothelial cells (Flt1-DsRed), resulting in the generation of Prox1-GFP/Flt1-DsRed (PGFD) mice. The inherent fluorescence of the blood and lymphatic vessels in these mice allows the direct observation of vascular structures in various organs via confocal and two-photon microscopy.^161^ With the publication of findings on the direct connection of CAVs with mLVs, PGFD mice are anticipated to play an even more vital role in further revealing the characteristics of mLVs in the CAV region. This advancement holds promise for addressing unresolved questions regarding the microstructural linkage between the glymphatic system and mLVs.

Advancements in the methodologies for observing mLVs in humans and animals are pivotal for advancing our understanding of the functions of mLVs within the CNS. Noninvasive techniques that have recently matured have the potential to decipher several enigmas pertaining to CSF drainage via mLVs. These include pinpointing the principal drainage sites of CSF within human mLVs, particularly whether they are situated at or beyond the superior sagittal sinus, especially posteriorly. This raises the question of whether dura-mater channels act as auxiliary pathways for CSF egress from the cranial cavity. Similarly, in a clinical setting such as chronic subdural hematoma, could scalp massage improve patient outcomes in a manner akin to CLN massages? There remains a lack of clarity as to why CSF drainage is greater in males than in females. Moreover, the difference in CSF drainage volume through mLVs across various disease models has not yet been determined. As observation techniques for mLVs evolve, these and other issues may be resolved, enhancing our comprehension of the variations in mLV drainage rates. Such insights are invaluable for formulating novel therapeutic approaches and drug delivery strategies.

Recent studies have shown that NIR imaging^123^ and photoacoustic imaging^124^ techniques can be used to observe dynamic drainage in mLVs in mice, but these modalities have not been reported in human mLV studies to date. NIR and photoacoustic imaging may become potential noninvasive methods for observing human mLVs in the future. Owing to equipment limitations and other factors in animal studies, ex vivo tissue staining remains the primary method for studying mLVs. As dorsal mLVs are more accessible, much of the research has focused on these vessels, but more reports pertaining to the basal mLVs and the NPLP are expected as technology matures. Given the lower image resolution and invasiveness of two-photon imaging, photoacoustic imaging is currently the preferred noninvasive method for observing mLVs in mice. With the advancement of various probe and tracer materials, NIR technology will be able to visualize mLVs directly soon without relying on Prox1 transgenic mice. As advancements in methods for observing mLVs in animal models continue to progress, the precise pathways of CSF drainage along with the clearance of macromolecules and cells through mLVs are anticipated to be increasingly elucidated. Understanding the routes taken by CSF and large biomolecules from the brain to mLVs will contribute to a deeper understanding of the mechanisms governing CSF drainage and neuroimmune interactions under both physiological and pathological conditions. Additionally, this knowledge will aid in the development of novel CNS drug delivery systems, potentially transforming our approach to treating a variety of neurological disorders.

## Functions of mLVs

**Fig. 6:**
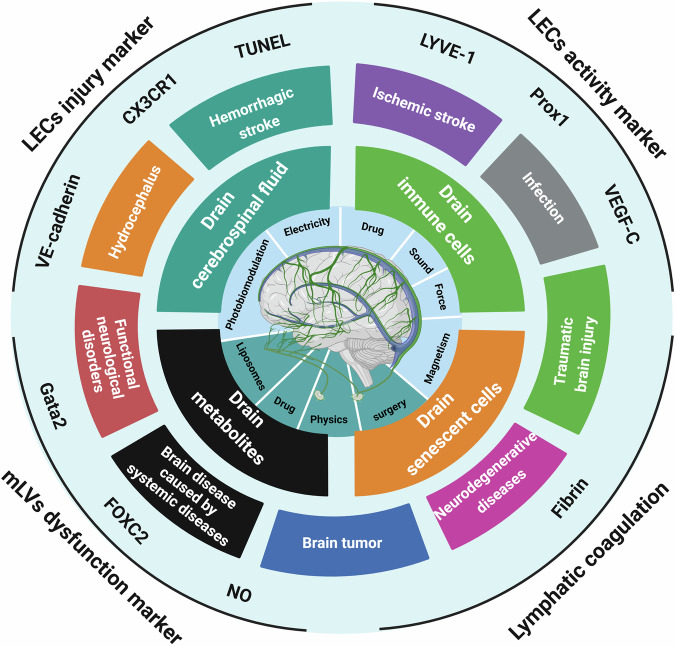
Promising target for fundamental research and preclinical strategies. The figure summarizes the promising targets for interventions directed at LECs injury and dysfunction, with a primary focus on LECs activity marker, LECs injury marker, mLVs dysfunction marker, and lymphatic coagulation. Schematic representation of the functions of the mLVs and associated diseases includes neurodegenerative diseases, TBI, hemorrhagic stroke, ischemic stroke, infections, tumors, functional neurological disorders, hepatic encephalopathy, and secondary hydrocephalus. Moreover, the illustration summarizes the current classification of interventions targeting mLV impairment. (Created with BioRender.com)

The lymphatic system is essential for maintaining fluid homeostasis and immune defense. Traditionally, lymphatic vessels have been implicated primarily in the pathogenesis of primary and secondary lymphedema. Recent studies, however, have linked lymphatic dysfunction to a spectrum of diseases, including cardiovascular disorders, glaucoma, inflammation, Crohn’s disease, hypertension, obesity, and atherosclerosis.^162^ In the context of CNS diseases, mLVs have emerged as key regulators. Recent research has underscored the importance of mLVs in conditions such as secondary hydrocephalus, intracerebral hemorrhage (ICH), neurodegenerative diseases, TBI, and CNS infections. Given the absence of prior studies categorizing the functions of mLVs, we delineated their drainage capabilities, which encompass the clearance of CSF, metabolic byproducts, senescent cells, and immune cells from the brain (Fig. 6). These findings underscore the critical importance of lymphatic system integrity for overall health and for the prevention and therapeutic management of diseases.

### mLV pathways in CSF drainage

Research on the drainage of CSF by CLNs dates back to the 1980s, revealing a multifaceted understanding of mLVs. Initially, studies such as those by Vera Quesada et al. in 1980 cannulated the cervical lymphatic vessels of rabbits and cats to quantify the proportion of CSF drained through this pathway. Their findings indicated that in rabbits, 30% of CSF drained via mLVs to dCLNs, whereas in cats, the estimated percentage was approximately 13%.^163^ In 1992, Cserr et al. reviewed several animal studies and suggested that approximately 50% of CSF drainage was mediated by dCLNs.^24^ Building on these perspectives, subsequent research by Knopf et al. in 1995 explored the role of mLVs in the drainage of large molecules and the function of mLVs in immune surveillance.^164^ A landmark study by Dr. Alitalo’s^16^ and Dr. Kipnis’s teams^17^ independently demonstrated the drainage of CSF to dCLNs by mLVs in mice. Probing the human applicability of these findings, Absinta et al. visualized mLVs in humans and primates via gadobutrol-enhanced lymphatic imaging with T2-FLAIR and T1-weighted MRI, providing a noninvasive method for observing mLVs in humans.^86^ These investigations have culminated in detailed structural analyses exemplified by Ahn et al. who reported that mLVs at the skull base had larger diameters and abundant protruding primary lymphatic vessel branches with typical oak leaf-shaped (button-like) junctions and lymphatic valves and lacked smooth muscle coverage, thus structurally favoring CSF drainage.^78^ In 2017, Ma et al. reported a significant decrease in CSF lymphatic outflow in aged mice compared with young mice, suggesting that the lymphatic system is a target for age-related neurological diseases.^23^ Recent advances demonstrated by Jacob et al. demonstrated the three-dimensional anatomy of mLVs in humans via real-time vessel-wall MRI after systemic injection of gadobutrol, providing high-level evidence for CSF drainage by mLVs.^80^ In 2023, Vera Quesada et al. reported a wider distribution of LECs in the human dura mater away from the venous sinuses.^84^ Subsequent studies by Shah et al. (2023) revealed LECs in human AGs, supporting the direct transport of CSF from the subarachnoid space to the mLV system.^87^ Additionally, in 2024, Yoon et al. identified the NPLP, a major hub for CSF drainage from mLVs to dCLNs, in the nasopharyngeal mucosa of macaques and mice, thereby clarifying the drainage pathways of intracranial mLVs.^82^

In summary, early studies in animals such as cats and rabbits suggested that mLVs account for approximately 50% of CSF drainage, with further evidence highlighting the crucial role of mLVs in various disease conditions. Intracranial mLVs represent a significant pathway for CSF outflow. The spine, as part of the CNS, also contributes to CSF drainage, with recent studies indicating that the remaining CSF (50%) drains from the spinal cord to the mediastinal, iliac, and sacral LNs^18,35,165^ or through perivascular spaces.^24^ Continued exploration is needed to elucidate the mechanisms underlying the relationship between CSF drainage dysfunction and the pathogenesis of neurological diseases, as well as to determine whether modifications or therapeutic targeting of these pathways could offer novel approaches for treatment.

### mLV drainage of metabolic byproducts

#### *mLVs* drainage of amyloid-*β*(*Aβ*) proteins

AD is a progressive neurodegenerative disorder characterized by the accumulation of Aβ protein, imposing a significant burden on patients and their families. Despite the substantial impact of AD, effective treatments remain elusive. The clearance of the Aβ protein is considered a promising therapeutic strategy for AD.^25^ In 2018, two independent groups reported that mLVs could facilitate the drainage of Aβ proteins, which is dysfunctional in both AD transgenic mice and aged mice.^39,166^ This finding was supported by Ma et al.’s 2017 findings, which indicated a marked decline in CSF lymphatic outflow in aged mice compared with that in younger mice, suggesting that mLVs can drain the Aβ protein and that this drainage capacity diminishes with age.^23^ Further evidence was provided by the research team led by Zhibin Yao in 2018.^28^ Their work revealed that an injection of VEGF-C promoted the proliferation of LECs and increased the drainage of Aβ, confirming the role of mLVs in Aβ protein clearance. These findings collectively underscore the potential of targeting mLVs as a therapeutic approach for AD^167^ and cerebral amyloid angiopathy.^168^

#### mLV drainage of tau proteins

AD is characterized by two principal neuropathological hallmarks: the extracellular accumulation of Aβ proteins and the intracellular aggregation of tau proteins. In addition to its role in AD, the tau protein is implicated in a spectrum of neurodegenerative disorders collectively referred to as tauopathies. These diseases include progressive supranuclear palsy, corticobasal degeneration, certain forms of frontotemporal dementia, and argyrophilic grain disease. The first study reporting that the meningeal lymphatic system drains the tau protein from the brain was performed by Cao et al. ^169^ who reported that dCLNs increased total and phosphorylated tau protein levels in the hippocampus of both WT mice and AQP4 null mice. The mLV drainage of the tau protein was confirmed by follow-up studies. In 2019, Patel et al. reported that the injection of tau-coupled tracers into the brain parenchyma revealed the presence of tau protein deposits within mLVs and CLNs.^40^ Moreover, Pu et al. reported that blockage of brain lymphatic drainage by an intracisternal injection of autologous blood resulted in the accumulation of tau protein as well as CD3^+^, CD4^+^, and CD8^+^ cells in the mouse brain.^170^ The involvement of mLVs in the clearance of the tau protein underscores the potential of the lymphatic system as a therapeutic target in individuals with tauopathies. The discovery that mLVs may facilitate the removal of tau from the CNS opens new avenues for research into treatments that could modulate this drainage pathway.

#### mLV drainage of α-synuclein

CSF drained by the lymphatic system is rich in α-synuclein (α-syn), a protein intimately associated with the pathogenesis of PD and neuroinflammation. The first article reporting mLV drainage of α-syn was published by Zou et al. in Transl Neurodegener,^171^ and the authors reported that the glymphatic influx of CSF tracers was reduced in A53T mice, accompanied by perivascular aggregation of α-syn and impaired polarization of aquaporin 4 expression in the substantia nigra. Cervical lymphatic ligation aggravated glymphatic dysfunction in A53T mice, causing more severe accumulation of α-syn, glial activation, inflammation, dopaminergic neuronal loss and motor deficits. Impaired meningeal lymphatic drainage was confirmed in patients with idiopathic PD by Ding et al. (2021). They also confirmed that in mice injected with preformed fibrils of α-syn, deposits of α-syn were present in mLVs, leading to delayed lymphatic drainage. This α-syn deposition was accompanied by a loss of tight junctions between the endothelial cells of the mLVs and increased meningeal inflammation.^37^ Building on these findings, in 2023, Liu et al. examined structural and functional changes in the dLNs of PD model mice (A53T mice). They reported that lymph node enlargement is closely associated with macrophage activation, which is induced by the drainage of oligomeric α-syn through mLVs, leading to peripheral inflammation in PD patients.^38^ In summary, these findings highlight the critical role of the lymphatic system in the clearance of α-syn and its implications for the pathophysiology of PD. The evidence suggests that impaired lymphatic drainage can exacerbate neurodegenerative processes and inflammation, suggesting potential targets for therapeutic intervention. The ongoing exploration of the involvement of the lymphatic system in neurodegenerative diseases continues to provide valuable insights into the mechanisms underlying these disorders and the development of novel treatment strategies.

#### mLV drainage of TDP-43 and glutamate

Intracranial accumulation of TAR DNA-binding protein 43 (TDP-43) and glutamate is directly implicated in the pathogenesis of amyotrophic lateral sclerosis (ALS). The mLV system facilitates the transport of solutes and the clearance of toxic substances from the brain. A pivotal study by Eisen et al. in 2024 demonstrated that the pathogenic mechanisms of ALS are associated with glymphatic and mLV system dysfunction, leading to impaired drainage of TDP-43 and glutamate.^33^ In essence, the findings of this research underscore the importance of the mLV system in the neuropathology of ALS, suggesting that disruptions in the clearance pathways for neurotoxic proteins and excitatory amino acids may contribute to disease progression. These findings advocate further investigation into the therapeutic potential of targeting lymphatic drainage in ALS, with the aim of ameliorating the accumulation of harmful substances in the CNS.

#### mLV drainage of cellular debris

The relationships between TBI-induced cerebral edema and the lymphatic system (including the glymphatic system and meningeal lymphatics) were investigated by Hussain et al. in 2023. Using transgenic mice expressing the calcium indicator GCaMP7 in cortical astrocytes and neurons, the research team observed dCLNs post-TBI and discovered that the cellular debris in these LNs originated from the cortex.^45^ In summary, the findings suggest that following CNS injury, the cellular debris can be drained through mLVs to the CLNs, potentially alleviating damage. The findings of this study highlight the importance of the lymphatic system in the clearance of postinjury byproducts and underscore the potential therapeutic value of increasing lymphatic drainage in the context of TBI.

Despite current reports indicating that the metabolic byproducts produced by mLVs include amyloid-β (Aβ) proteins, tau proteins, α-synuclein, TDP-43, and glutamate cellular debris, it remains unclear how these metabolites enter mLVs. Furthermore, it is important to investigate whether there are any upper limits to their molecular weight and diameter. In-depth research into these issues may provide potential therapeutic targets for the targeted treatment of CNS diseases, including neurodegenerative disorders.

### mLV drainage of senescent cells

#### mLV drainage of senescent astrocytes

The elimination of senescent astrocytes via mLVs is a key process for maintaining homeostasis within the CNS, particularly during aging. In 2022, Li et al. conducted a study on a mouse model of AD and discovered that perivascular spaces and mLVs constitute a functional pathway for the clearance of senescent astrocytes from the aging brain. The chemokine CCL21 was identified as a mediator of the drainage of senescent astrocytes through mLVs.^36^ Therefore, senescent astrocytes are cleared via CCL21-mediated drainage through mLVs to CLNs, a process potentially vital for CNS homeostasis and a prospective target for treating age-related neurodegeneration. However, the precise mechanisms regulating the interaction between CCL21 and senescent astrocytes in the context of mLV drainage and the overall efficiency of this clearance pathway in the diseased state warrant further investigation. These insights could unveil novel strategies for enhancing the clearance of senescent cells and potentially decelerate the progression of neurodegenerative diseases.

#### mLV drainage of red blood cells

CSF-derived red blood cells (RBCs) play a pivotal role in the pathogenesis of hemorrhagic stroke. In 1979, Oehmichen et al. reported that marker-labeled erythrocytes, lymphocytes, and/or peritoneal macrophages injected into the brain were subsequently identified in CLNs, indicating the presence of all labeled cell types.^172^ Further research by Oehmichen et al. in 1983 corroborated these findings by detecting the drainage of intracerebrally injected erythrocytes into CLNs.^173^ Chen et al. reported a significant increase in RBCs within CLNs and mLVs following subarachnoid hemorrhage (SAH), suggesting that mLVs facilitate the drainage of CSF-extracted RBCs to CLNs.^174^ Subsequent research by Li et al. revealed that exposure to NIR light at 1267 nm and 9 J/cm^2^ increases lymphatic drainage and clearance in neonatal rats via NO-mediated regulation of mLV tension, thereby accelerating RBC drainage post-IVH.^52^ The mLV system is responsible for the clearance of macromolecules and pathogenic substances from the CNS. Tsai et al. assessed the drainage function of the mLV system using CSF tracers and PKH-26-labeled RBCs, with findings published in Stroke.^54^ These results indicate that mLV generation and lymphatic drainage are increased in the late stages of ICH and that early enhancement of mLV function is beneficial for ICH recovery. Wang et al. reported that the administration of dobutamine after SAH promoted the clearance of RBCs and their degradation products via mLVs, thereby alleviating early neurological deficits.^53^ Yuan et al. discovered that RBCs are drained into CLNs in CSDH model rats via mLVs, with CSDH inducing ERK1/2 dephosphorylation in mLV endothelial cells, leading to basal mLV disruption and impaired drainage. Atorvastatin ameliorated post-SDH injury by improving the structure of basal mLV endothelial cell junctions.^47^

In conclusion, mLVs paly a fundamental role in clearing RBCs and their metabolic byproducts from the CSF, which is paramount for alleviating neurological deficits following hemorrhagic stroke. Modulating mLVs to expedite RBC drainage represents a potential therapeutic avenue following hemorrhagic stroke. However, unanswered questions remain regarding the detailed molecular mechanisms underlying mLV-mediated clearance of RBCs and the optimal therapeutic window for interventions. Further studies are needed to elucidate the dynamics of the mLV response in different stages of hemorrhagic stroke and to tailor therapeutic approaches to individuals’ specific pathological conditions.

### mLVs involved in the immune response

Emerging evidence suggests that there is a complex interplay between the CNS and the peripheral immune system, culminating in an intricate immune network.^175–177^ mLVs play a crucial role in immune surveillance within the CNS, facilitate the transport of immune cells to the periphery and modulate peripheral immunity.^178^ Conversely, peripheral diseases can impact the function of mLVs.^58,175,179^ Thus, in addition to directly regulating mLVs, current research is exploring the cutting-edge frontiers of drug delivery via CLNs or intranasal routes. This section provides an overview of the latest advancements in our understanding of the relationships between mLVs and peripheral immunity, between mLVs and parenchymal border macrophages (PBMs) and between mLVs and CNS immunity.

#### Relationship between mLVs and peripheral immunity

During disease states, the mLV system transports immune cells from the CNS to the periphery, establishing interactions with the peripheral immune system.^180–184^ By antigen presentation, mLVs mediate the entry of central immune cells such as dendritic cells (DCs), B lymphocytes, T cells, and neutrophils into the peripheral immune system, thus inducing autoreactive T-cell responses^185,186^ and modulating peripheral immunity.^167^

##### mLV drainage of DCs

DCs are the major professional antigen-presenting cells in the human body and possess the ability to capture, process, and present antigens. DCs play crucial roles in activating and regulating populations of T cells and B cells. In 2018, a study by Louveau et al. revealed the presence of DCs in mLVs. Subsequent research by this group involved injecting tracer-labeled DCs into the cisterna magna to observe their drainage into mLVs and dCLNs, and the findings indicated that DCs can be drained via the mLV system and participate in central immune responses.^132^ In conclusion, DCs from the CNS can be drained to CLNs via mLVs and engage in immune crosstalk with peripheral immune regulators. However, the precise roles and mechanisms by which DCs operate within this system remain to be fully elucidated. Future research should focus on deciphering the detailed functions of DCs in immune surveillance and on the development of targeted interventions that leverage their unique role within the mLV pathway.

##### mLV drainage of B cells

The central role of B lymphocytes in maintaining immune system homeostasis is widely recognized. As abnormal B-cell function can potentially trigger autoimmune and neurodegenerative diseases of the CNS, its contribution to the pathogenesis of diseases has garnered increasing attention over the past few decades. Particularly in studies of experimental autoimmune encephalomyelitis (EAE) and multiple sclerosis (MS) induced in laboratory animals, B lymphocytes have been found to play dual roles: B cells not only contribute to the pathogenic progression of the disease but also participate in disease regulation.^187^ Researchers such as Brioschi have recently investigated the migration pathways of B lymphocytes in the CNS, confirming that B lymphocytes present in the meninges can indeed migrate to CLNs through the transverse and sigmoid sinuses located in the dura mater via a drainage mechanism.^188^ Despite these advances, unanswered questions persist regarding the exact functional roles and mechanistic pathways through which B lymphocytes from the CNS migrate to and interact within the peripheral immune system. Future investigations should endeavor to unravel the nuances of B-cell involvement across different stages of CNS-related disease development and the implications for therapeutic targets.

##### mLV drainage of T cells

Neuroinflammatory diseases, such as MS, are characterized by the infiltration of autoreactive T cells into the brain. However, the mechanisms by which T cells acquire their encephalitogenic phenotype and trigger disease remain unclear. The first article reporting mLV drainage of T cells was published by Louveau et al in 2015.^17^ In 2018, a study by Louveau et al. revealed that immune cells can enter draining LNs in a CCR7-dependent manner. Unlike other tissues, endothelial cells within mLVs do not proliferate during inflammation and exhibit unique transcriptional features. Research by this group revealed that ablating mLVs in an animal model of MS reduces pathology and diminishes the inflammatory response of brain-reactive T cells. These findings indicate that mLVs control inflammatory processes and immune surveillance in the CNS.^132^ In their 2018 review, Rua et al. described how the location and activation state of meningeal immune cells can influence the homeostasis of the CNS, leading to neurological diseases, but these cells also have the capacity to protect the CNS from pathogen invasion.^189^ In 2021, a study by Da Mesquita et al. revealed reduced CCR7 expression in the meningeal T cells of AD model mice, in which CCR7 deletion led to increased neuroinflammation, microglial activation, and increased Aβ deposition in the brain. The findings of this study highlight the potential key role of CCR7-mediated T-cell immunity in the pathogenesis of AD.^190^ In 2023, Rustenhoven et al. reported that age-related changes in meningeal immunity underlie damage to mLVs. Single-cell RNA sequencing of samples of endothelial cells from mLVs from aged mice revealed their response to IFNγ, which increased in aged meninges due to T-cell accumulation. Compared with defects in aged mice, overexpression via adeno-associated virus (AAV)-mediated delivery weakened CSF drainage in young mice and chronic elevation of meningeal IFNγ. Degradation or inhibition of IFNγ improved the function of mLVs in age-related damage. These data suggest that regulating meningeal immunity may provide a feasible approach to normalize CSF drainage and alleviate the neural functional deficits associated with impaired waste clearance.^191^ In summary, T cells are key participants in neuroinflammatory conditions and aging-related CNS abnormalities, fundamentally through their trafficking via mLVs. Despite these insights, questions about how the molecular and cellular mechanisms of mLVs contribute to these diseases persist and how we can harness this knowledge for therapeutic interventions remain to be elucidated.

##### mLV drainage of neutrophils

Neutrophils perform a wide range of immune functions, including active phagocytosis of invading microorganisms, increased recruitment of local immune cells, and passive modulation of cytokine and growth factor secretion at sites of inflammation.^192^ A study by Cugurra et al. published in 2021 revealed the presence of a population of monocytes and neutrophils in the meninges of mice, which were not supplied by blood but rather by the adjacent cranial and vertebral marrow. These findings necessitate a reevaluation of how immune cells infiltrate the CNS during injury and autoimmune responses, potentially providing insights for future therapeutic approaches utilizing meningeal immune cells.^193^ In IVH model mice, Zhang et al. reinfused CFSE-labeled neutrophils into the CSF via cisterna magna injection and observed morphologically intact neutrophils in both mLVs and dCLNs.^46^ These results indicate that neutrophils from the CNS can be recruited to mLVs and drain CLNs following IVH, where they play crucial roles in post-IVH inflammatory responses. In conclusion, cranial neutrophils can be recruited to mLVs through chemokine regulation in autoimmune diseases and hemorrhagic stroke, subsequently draining to CLNs and exerting diverse functions in diseased states. The specific mechanisms underlying the ingress and egress of neutrophils into and out of mLVs remain unclear. Moreover, whether the movement of neutrophils through mLVs is unidirectional or bidirectional constitutes an unresolved question in the field.

#### Relationships between mLVs and PBMs

Macrophages are key players in maintaining tissue homeostasis. In 2022, Drieu et al. identified a subset of PBMs in mice characterized by high expression levels of CD163 and LYVE-1, demonstrating that LYVE-1^+^ PBMs regulate arterial pulsatility, which drives CSF flow. This study provides evidence that PBMs modulate the dynamics of CSF flow, positioning them as novel cellular regulators of CSF hemodynamics. These findings suggest potential pharmacological targets to ameliorate the brain clearance deficits associated with aging and AD.^194^ In summary, LYVE-1^+^ and CD163^+^ macrophages within the brain parenchyma, termed PBMs, play a significant role in regulating the arterial pulsatility that influences CSF flow. However, the relationship between these peripheral macrophages in the brain parenchyma and LYVE-1^+^ macrophages produced under disease conditions remains unclear. The evidence suggests that mLVs can be modulated in certain brain pathologies, including aging, AD,^23,39^ PD, and/or TBI. Although previous researchers have not observed lymphatic vessels in the meninges or brain parenchyma via whole-brain immunofluorescence staining techniques, a 2023 study by Chang et al. confirmed the existence of lymphatic vessels deep within the brain parenchyma. These researchers described the characteristics of mLVs in the cortex, cerebellum, hippocampus, midbrain, and brainstem of mice subjected to chronic psychosocial stress. Research has shown that deep brain tissues in mice can generate new mLVs in response to stressful life events. While the brain parenchyma physiologically lacks a lymphatic system, pathological proliferation may be associated with the levels of vascular endothelial growth factor-C (VEGF-C).^195^ In 2019, Chen et al. reported that cerebral ischemia induced rapid growth of mLVs toward the injured parenchyma in a zebrafish model of cerebral ischemia, thereby alleviating edema, and that mLVs underwent apoptosis and clearance after the regeneration of brain blood vessels.^196^ In summary, recent studies in zebrafish and mouse models have shown that mLVs can proliferate in deep brain tissues under conditions such as ischemic diseases and chronic stress-related disorders. Another article^197^ reported that “detailed analysis for the presence of LECs within the CNS using different reporter mice combined with immunostaining for LEC markers did not reveal evidence for these cells within the parenchyma. Only a few isolated LECs were observed within the pial meninges, as described previously^197^”. In this study, the author suggested that LYVE1^+^ cells are perivascular macrophages rather than LECs in the brain parenchyma. In summary, the existence of mLVs within the parenchyma of the mammalian brain is currently a matter of debate and requires further research for clarification. Furthermore, the connection between LYVE-1-positive cells in newly discovered AGs and peripheral macrophages is poorly understood. These areas warrant further investigation to elucidate the roles of PBMs in CNS physiology and pathology.

#### Relationship between mLVs and central immunity

The hubs involved in the interaction between peripheral immunity and the CNS through mLVs include the mLV-CLN axis, the cribriform plate, and the skull bone marrow. After CNS injury, brain-derived antigens, injury metabolites (e.g., cellular debris, proteins, DNA/RNA), and damage-associated molecular patterns (DAMPs) not only elicit central immune responses but also trigger peripheral immune reactions, leading to the migration of peripheral immune cells to the CNS.^198^ Proteins that significantly affect peripheral immunity include HMGB1,^199^ S100^200^, and ATP.^201^ The peripheral immune system maintains surveillance over the CNS, not only by recognizing external pathogens but also by playing a critical role in sterile CNS injuries such as ischemic/hemorrhagic stroke and TBI.^176^ Following ischemic stroke, peripheral organs including the intestine,^202^ bone marrow,^203^ thymus,^203^ adrenal glands,^203^ gastrointestinal tract,^203^ lungs,^204^ and spleen^205^ mediate and regulate interactions with the brain through circulatory pathways to modulate poststroke inflammation.^206,207^ The regulation of these interactions by the mLV‒CLN axis,^208,209^ along with dysregulation of the neuroendocrine and autonomic nervous systems, also plays a significant role in central and peripheral immune modulation.^210^ For example, a study by Esposito et al. in 2019 reported the proliferation of LECs in the CLN of rats with cerebral ischemia and the rapid activation of macrophages within 24 h in the CLN. Blocking VEGFR3 in the CLN decreased lymphatic endothelial activation, and proinflammatory macrophage levels and reduced cerebral infarction. Interestingly, surgical removal of mouse CLNs significantly diminished the infarct size after focal cerebral ischemia.^211^ This finding suggests that the CNS also assumes a role when peripheral trauma responses occur. In another 2024 study, Lee et al. reported that the rupture of mLVs improved spatial memory function in the late phase of ischemic stroke. The number of immune cells infiltrating the brain, including neutrophils, monocytes, T cells, and natural killer cells, decreased after cerebral ischemia‒reperfusion and mLV destruction.^209^ In an MS mouse model, ablating mLVs has been proven to suppress T-cell inflammatory responses and mitigate CNS damage.^132^ Furthermore, a 2024 study by Zhu et al. revealed that in aging individuals and patients with neurodegenerative diseases, surgeries could induce excessive neuroinflammation and PND.^212^ These results indicate that CLNs, as hubs between the central and peripheral immune systems, can amplify central immune responses globally, thereby affecting peripheral immunity, and vice versa.

Emerging research has underscored the critical role of neuroinflammation-induced lymphangiogenesis near the cribriform plate in CNS homeostasis. Specifically, drainage of CNS-derived antigens and immune cells is facilitated by lymphangiogenesis near the cribriform plate, as indicated by recent findings.^213–215^ Moreover, a 2019 study by Hsu et al. revealed that increased lymphangiogenesis near the cribriform plate aids in the management of inflammation-induced fluid accumulation and immune surveillance.^213^ In addition to this body of knowledge, Fitzpatrick et al. in 2024 discovered that lymphatic structures adjacent to the dural venous sinuses, termed dural-associated lymphoid tissues, are capable of sampling antigens and rapidly bolstering humoral immune responses following localized pathogenic assaults.^216,217^ These findings suggest that the cribriform plate serves as one of the hubs for delivering peripheral signals to mLVs, particularly highlighted by studies on intranasal drug administration modulating central immunity, which empirically underscore this point.^218^ Additionally, Mäkinen T suggested that mLVs may also promote immune tolerance^219^ (i.e., suppression of immune responses to recognized substances), similar to lymphatic vessels in LNs.^220^ This question warrants further investigation.

To date, the origin of bone marrow-derived immune cells present in the brain remains enigmatically underexplored. For example, Brioschi et al. (2021). reported that in adult mice, meningeal B cells are derived mainly locally from the calvaria. B cells reach the meninges from the calvaria through specialized vascular connections. In contrast, a subset of antigen-experienced B cells that populate the meninges in aging mice are bloodborne.^188^ Furthermore, the study conducted by Mazzitelli et al. published in 2022 demonstrated that CSF permeates into the cranial bone marrow through dural channels, where it influences a variety of cells within the bone marrow microenvironment. In the context of disease-related damage, CSF-derived signals increase the generation of cells bone marrow cells and their dissemination into the meningeal space.^91^ This finding elucidates a CSF-based mechanism of communication between the CNS and bone marrow, which plays a role in the regulation of CNS immune responses.^91^

With advancements in research on mLVs and peripheral immunity, lymph node-targeted drug delivery has become a focal point of research for the modulation of intracranial mLVs. Drug delivery targeting the LNs is a promising approach to improve the efficacy of immunotherapy for intracranial tumors.^221^ For example, in 2024 Zhao et al. achieved a 44-fold increase in drug uptake in the brain following the administration of indocyanine green (ICG)-loaded PLGA nanoparticles through a subcutaneous (s.c.) injection at the neck near a local LN compared with intravenous injection, effectively treating glioblastoma in mice through photodynamic therapy.^222^ This approach facilitated effective photodynamic therapy for glioblastoma in a murine model.

Additionally, the success of lymph node-targeted drug delivery^221^ and therapies such as autologous tumor lysate-loaded dendritic cell vaccine (DCVax-L)^223^ has prompted reflection on the failure of chimeric antigen receptor T (CAR-T) therapy: the number of T cells in the periphery is less than the number that can infiltrate the tumor microenvironment.^222^ The discovery of the ocular lymphatic system^90^ and the eye‒brain‒immunity connection,^89^ along with advances in intranasal^218^ and subcutaneous (s.c.) injection near a local LN^221^ for the treatment of glioblastoma, indicate an evolution in the understanding of brain immunity from “immune privilege” to “distinct immunity”. Interestingly, studies by Esposito et al.,^211^ Yang et al.,^48^ and Lee et al.^209^ seem to contradict previous research where the ligation of dCLNs typically aggravated intracranial inflammatory responses,^54^ a discrepancy worth considering. The role of dCLNs in disease states may be more complex than realized; on the one hand, they serve as an exit pathway for cranial products, with posthemorrhagic stroke leading to reduced drainage and hence ligation exacerbating the injury. On the other hand, CLNs are a venue for central‒peripheral immune interactions, where in specific models, such as postischemic stroke, peripheral immune cells are activated, and these systemic responses can amplify brain damage. Therefore, several key research directions may be pertinent for the future. These include further exploration of intranasal and CLN drug delivery mechanisms, detailed examinations of the complex ocular–brain and cranial immunity interplay and fine-tuning our strategies to effectively modulate mLVs. There is a need for a deeper understanding of how these disparate elements contribute to or hinder brain immunity and treatment outcomes, especially for CNS diseases. Addressing these unresolved questions and embracing novel perspectives could dramatically alter therapeutic approaches and improve prognoses for patients with intracranial pathologies.

## Molecular mechanisms underling mLV injury

The study of the lymphatic system has a long history,^224^ as the lymphatic system plays a crucial role in maintaining the homeostasis of the extracellular fluid and immunity,^225^ whereas the aberrant formation of lymphatic vessels is associated with various diseases.^226^ However, many mechanisms of the lymphatic system remain to be further investigated.^227^ Research into lymphangiogenesis and injury cannot be separated from specific markers of LECs: VEGFR-3, VEGF-C/D, PROX1, podoplanin, LYVE-1, and FOXC2.^228^ In addition to relying on glycolysis, LECs also utilize other metabolic pathways such as fatty acid β-oxidation, ketone oxidation, mitochondrial respiration, and lipid droplet autophagy to support lymphangiogenesis.^229,230^ Moreover, the development of mLVs is also influenced by lipid metabolism, extracellular vesicles, and fluid shear stress.^231^ In this context, we review the previously reported molecular mechanisms of mLV injury and, in conjunction with peripheral lymphatic research, discuss potential molecular pathways of mLV injury for future exploration.

### VEGFC-VEGFR3 signaling pathway

The vascular endothelial growth factor C/vascular endothelial growth factor receptor 3 (VEGFC‒VEGFR3) signaling pathway has long been considered the primary molecular driver of lymphangiogenesis.^75,231^ A deficiency in VEGFC leads to the absence of lymphatic development,^232–235^ and the loss of VEGFR3 results in primary lymphedema.^236^ Early research revealed the crucial role of calcium binding EGF domain 1 (CCBE1) in the embryonic development of zebrafish and mice.^237–240^ Subsequent studies indicated that CCBE1 is involved in the maturation of full-length VEGFC,^241–243^ which is necessary for the initial steps of lymphatic development.^244–247^ CCBE1 also contributes to the migration of LECs.^248^ Research by Ocskay et al. published in 2024 revealed that inducible deletion of CCBE1 led to postnatal impairment of meningeal lymphatic vessel development and decreased macromolecule drainage to dCLNs.^249^ This study revealed that the degeneration of dorsal mLVs following the loss of CCBE1 was similar to that in aged mice, suggesting that CCBE1 could be a potential therapeutic target to prevent the age-dependent regression of mLVs. Research by Boisserand et al. published in 2024 explored the impact of overexpressing VEGF-C on CSF drainage and the outcome of ischemic stroke in mice and demonstrated that VEGF-C administration promoted various vascular, immune, and neural responses, ultimately preventing acute ischemic stroke-induced neurological damage.^250^ Furthermore, a 2024 study by Kovacs et al. reported that VEGFC treatment increased CSF outflow during toxoplasmosis brain infection but did not alleviate brain edema.^57^

**Fig. 7:**
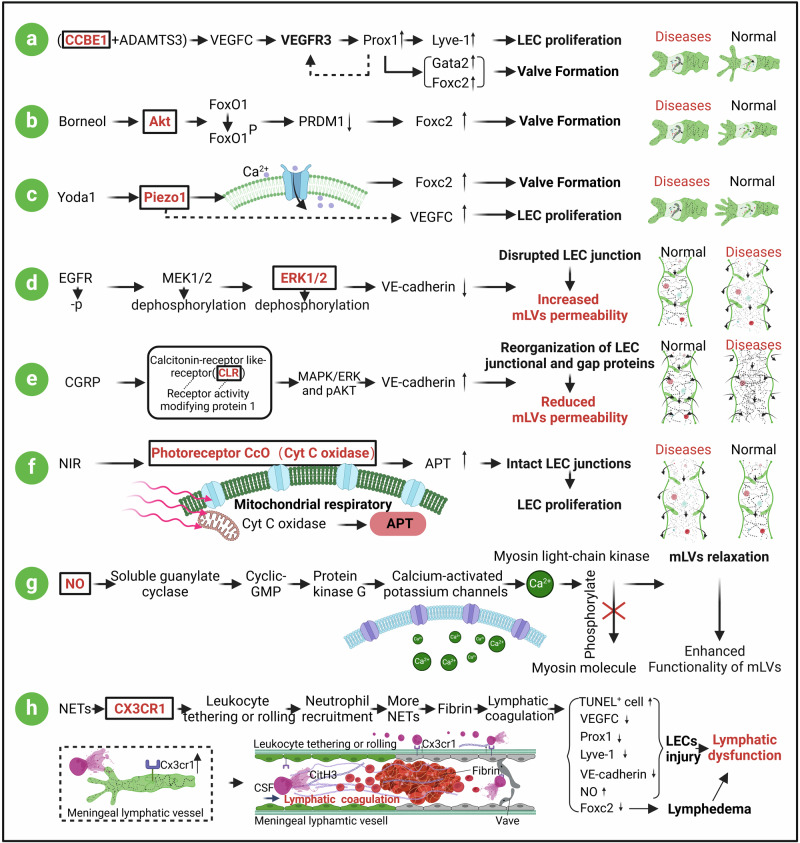
Molecular pathways involved in the mechanism of mLV injury. **a** CCBE1 is involved in the processing of VEGFC,^241–243^ which is essential for the initial steps of lymphangiogenesis. The absence of CCBE1 and VEGFC leads to impaired development and dysfunction of meningeal lymphatic vessels.^249^ **b** FOXC2 is one of the main promoters of lymphatic valve development, while the forkhead transcription factor FOXO1 acts as an inhibitor of lymphatic valve formation and maintenance in LECs. Elevated FOXO1 is associated with reduced FOXC2, and Akt-mediated phosphorylation leads to FOXO1 inactivation, thereby facilitating lymphatic valve formation.^264,265^ **c** Piezo1 controls mLV drainage through two main mechanisms: it enhances the expression of Foxc2 and augments interstitial flow and functional drainage by facilitating VEGF-C expression, VEGFR3 activation, and lymphatic endothelial cell proliferation via integrin-mediated interactions with the extracellular matrix.^282^ **d** ERK1/2 signaling has been established as a participant in lymphangiogenesis,^290,291^ where EGFR dephosphorylation mediates the subsequent dephosphorylation of MEK1/2 and ERK1/2. This dephosphorylation of ERK1/2 may lead to a reduction in VEGFR3 and connexin expression, resulting in discontinuation of basal mLVs and impaired mLV drainage.^47^ **e** CGRP-triggered CLR signaling pathway activation led to the reorganization of LEC junctional and gap proteins, culminating in impaired lymphatic drainage function.^316^ **f** CcO-activated through PBM augments mitochondrial respiration and ATP synthesis efficiency in LECs, fostering enhanced cellular vitality and facilitating functional repair.^328,329^ Concurrently, CCO-induced NO production promotes ATP generation and is associated with ROS signaling pathways. **g** NO acts as a vasodilator by stimulating soluble guanylate cyclase, elevating cyclic-GMP, which activates protein kinase G, opens Ca2^+^-activated K^+^ channels, and promotes Ca2^+^ reuptake, inhibiting myosin light-chain kinase activity, and inducing lymphatic vessel relaxation.^52,343^ **h** CX3CR1 mediates leukocyte recruitment to form lymphatic thrombi post-IVH, leading to LEC injury and malfunction of mLVs.^46^ (**a**–**h**: Created with BioRender.com)

In summary, the VEGFC‒VEGFR3 signaling pathway is highly important for the formation of mLVs. Methods to regulate this pathway include exogenous supplementation with VEGFC/CCBE1 or inhibition of VEGFR3 (Fig. 7a). Increasing VEGFC levels typically involves direct administration into the cisterna magna or lateral ventricle as a common method to improve mLV function^28,62^ and the use of AAV-mediated VEGFC overexpression.^39^ Blocking VEGFR3 involves downregulating VEGFR3 receptor proteins on mLV endothelial cells, an effective approach to induce meningeal lymphatic vessel dysfunction, also known as lymphatic ablation.^75,251^ Research concerning the role of CCBE1 in mLVs is scarce, and more investigations and evidence are needed to clarify its regulatory effects on mLVs. Intriguingly, Kovacs et al. utilized VEGFC to regulate dysfunctions in mLVs caused by toxoplasmosis but could not improve brain edema, a phenomenon that merits further analysis. However, whether the simultaneous regulation of mLVs and lymph-like vessels (such as increasing the activity of marginal macrophages) can ameliorate brain edema following toxoplasmosis infection remains unclear. Reports also indicate that exogenous or excessive VEGFC can result in the incomplete, immature, and aberrant formation of mLVs.^252^ Therefore, investigations of the most beneficial method for increasing VEGFC levels in mLVs are worth pursuing.

### Lymphatic valve development signaling pathways

The development of lymphatic valves is regulated by a variety of hemodynamic factors^253–255^ and is closely associated with key regulators such as Prox1.^256^ The transcriptional regulators that drive valve development and are modulated by shear stress include Foxc2^257–259^ and Gata2.^260^ (1) Gata2: Research by Feng et al. published in 2024 revealed that genes associated with lymphatic vessel development and function, such as Gata2 and Foxc2, are downregulated, suggesting that *Listeria monocytogenes* (LM) infection may impact cellular polarization and valve development.^58^ LM bacterial infection impairs the mLV-mediated drainage of macromolecules. (2) FOXC2: FOXC2 is a specific marker for lymphatic valves. Studies have shown that reduced expression of FOXC2 contributes to the occurrence of mLV edema.^46^ Deletions of the human FOXC2 gene have been shown to cause lymphedema-distichiasis (LD) syndrome.^261,262^ Investigations using AD model mice^128^ and mouse disease models with IVH^46^ have reported reduced levels of FOXC2 expression. Research by Ye et al. published in 2024 revealed that borneol (BO) is an enhancer of mLV lymphatic valve formation and can prevent or repair damage induced by toxic Aβ42.^263^ The mechanism involves Akt phosphorylation, which mediates BO action. FOXC2 is one of the most well-characterized promotors of lymphatic valve development. Research has indicated that the forkhead transcription factor FOXO1 is an inhibitor of mLV lymphatic valve formation and maintenance and is correlated with reduced FOXC2 expression (Fig. 7b). However, Akt phosphorylation leads to FOXO1 inactivation, resulting in the activation of the transcriptional repressor PRDM1.^264,265^

However, the molecular signaling pathways involved in lymphatic valve development or regulation remain unclear.^266^ For example, the relationships between GATA2 and Prox1 and the regulatory elements mediating the impact of shear stress on GATA2 expression have yet to be determined.^266^ The upstream regulatory relationship between GATA2 and PROX1 is also poorly understood.^257,267^ In addition to Prox1, the development of lymphatic and venous valves is influenced by many shared molecular pathways.^268^

### Piezo1 signaling pathway

Piezo1 is a mechanosensitive ion channel protein that is associated with a variety of physiological processes including the regulation of osmotic pressure, blood pressure, and epithelial and vascular development,^269^ and regulates the development of lymphatic valves^270–272^ and the proliferation of LECs.^273^ Loss of Piezo1 can lead to generalized lymphatic dysplasia of Fotiou (GLDF) and autosomal dominant dehydrated hereditary stomatocytosis with or without pseudohyperkalemia and/or perinatal edema (DHS).^274^ Yoda1 is a small-molecule activator of Piezo1.^275,276^

Craniosynostosis (CS) is characterized by the premature fusion of one or multiple cranial sutures, leading to deformities in cranial bone development and associated increased intracranial pressure (IICP). Primary expansion of the skull occurs at the sutures between bones, which is also concurrent with the development of mLVs.^277^ In 2022, Xiang et al. reported that IICP reduces the drainage of dCLNs and the function of dorsal mLVs while enhancing lymph flow toward the sacral LNs from the spinal cord, impacts that are associated with the severity of IICP.^278^ Subsequently, studies by Goodman et al. (2024) reported that acute antihypertensive treatment could improve CSF clearance rates, indicating that reducing blood pressure to increase CSF clearance may have therapeutic potential for diseases characterized by CSF circulatory dysfunction.^279^ In 2023, Stevenson et al. demonstrated that restoring mLV function improved cranial pressure and neurocognitive function in individuals with craniosynostosis.^280^ Research by Aspelund et al. conducted in 2024 revealed that the activation of the mechanosensor Piezo1 by Yoda1 could restore mLV function and CSF perfusion in models of CS and in aged mice.^281^ The mechanism by which Piezo1 regulates mLV drainage includes promoting the expression of Foxc2, on the one hand, increasing interstitial flow and functional drainage can stimulate VEGF-C expression, VEGFR3 activation, and cell proliferation through integrin-dependent interactions between LECs and the extracellular matrix^282^ (Fig. 7c). ‘Gain-of-fluid’ experiments have shown that increasing the amount of interstitial fluid elongates LECs and increases both VEGFR3 phosphorylation and LEC proliferation.^282^ Concurrent studies also indicate that Piezo1-regulated mLV drainage improves the accumulation of CSF.^283^

The aforementioned results suggest that intracranial pressure (ICP) and blood pressure correlate with mLV drainage. Importantly, an elevated ICP commonly occurs in conditions such as hydrocephalus, TBI, ICH, IVH, and posttumor complications. Whether the observed decrease in mLV drainage in models of these diseases is related to the regulation of Piezo 1 remains unclear. Recent studies on the clearance rate of metabolic waste products during sleep have also made groundbreaking advances. Sleep serves the active function of clearing metabolites and toxins from the brain. Previous investigations have indicated that enhanced clearance occurs during anesthesia as well.^131^ In 2024, Miao et al. reported that during sleep and anesthesia, the clearance ability of the brain does not increase but rather decreases, with removal rates decreasing by approximately 30% and 50%, respectively.^284,285^ This conclusion significantly challenges the findings of Xie et al. published in 2013, who suggested that natural sleep or anesthesia was associated with a 60% increase in the interstitial space, leading to a significant increase in the convective exchange between CSF and interstitial fluid^131^ and thereby facilitating the removal of metabolic waste.^286^ The study by Miao et al. indicated that the increased dye penetration observed during sleep or anesthesia might not be due to increased ingress rates but rather a decrease in clearance rates. Whether the reduced clearance rate during sleep is related to blood pressure and Piezo 1-mediated mLV drainage is currently unclear. Blood pressure and heart rate both decrease during sleep; could Piezo 1 regulation also have a threshold range, where too high an ICP causes a decline in mLV drainage, and likewise a reduction in blood pressure to a certain threshold also causes a decrease in mLV drainage? This field warrants further investigation.Sleep disorders are common in elderly people, and with increasing age in mice, cranial perfusion decreases.^287^ The relationships between sleep disorders and mLV drainage or the Piezo 1 signaling pathway remain to be elucidated. Recent studies on RCVS also revealed a correlation between mLV drainage and vascular constriction control.^287^ New research suggests that a decrease in mLV drainage is also related to functional neurological diseases, including syncope; however, the relationships among these ailments, blood pressure regulation, and Piezo 1 remain obscure. The interaction between the skull and brain is not yet fully understood, but in 2023, Ma et al. discovered that the skull functionally integrates with the brain through mLVs, which are impaired in individuals with craniosynostosis and can be restored through VEGF-C-driven lymphatic activation via skull progenitor cells.^288^ Considering the Lvs found by the authors in the skull, the relationship between mLVs and Lvs, as well as whether patients with Gorham–Stout disease (GS) have concurrent developmental aberrations in skull Lvs, remains unclear and is worth further exploration.

### ERK1/2 signaling pathway

ERK1/2 are dual-specificity kinases that participate in various biological processes, including transcription, proliferation, and cell adhesion.^289^ The ERK1/2 signaling cascade has been proven to be involved in lymphangiogenesis.^290,291^ The inactivation of ERK1/2 has been shown to disrupt intercellular junctions, characterized by decreased expression of VE-cadherin.^292,293^ Endothelial junctions are crucial for maintaining the integrity of lymphatic vessels,^294^ and the disruption of these junctions can lead to dysfunction in mLV drainage.^191^ VE-cadherin plays a vital role in the maintenance of lymphatic vessel integrity.^295,296^ Research by Meng et al. published in 2023 revealed that Efnb2-Ephb4 signal transduction can inhibit Erk activation in valve-forming cells to promote valve specification.^297^ A study by Yuan et al. published in 2024 revealed that SDH-induced dephosphorylation of ERK1/2 in meningeal LECs leads to interruptions in basal mLVs and impaired mLV drainage^47^ (Fig. 7d). The mechanism involves SDH-mediated dephosphorylation of EGFR, which further mediates the dephosphorylation of MEK1/2 and ERK1/2. Previous studies have indicated that ERK activation induces SOX18 and PROX1 expression.^290^ Additionally, ERK1/2 dephosphorylation might lead to decreased expression of VEGFR3 and cyclin.^298,299^

In summary, ERK1/2 may be key molecules involved in mLV damage, with mechanisms involving the VEGFR3 signaling pathway. While past research has shown that ERK1/2 are upstream of SOX18—whose deficiency has been proven to cause hypotrichosis–lymphedema-telangiectasia (HLTS) syndrome—the involvement of SOX18 in the damage mechanisms of mLV damage remains unclear.

### CGRP-CLR/RAMP signaling pathway

Calcitonin gene-related peptide (CGRP) is a potent vasodilatory neuropeptide released from trigeminal C-fibers during migraine that controls pain through the regulation of CSF outflow and neuroinflammation^300^ and is a neurotransmitter in both the central and peripheral nervous systems.^301,302^ The CGRP receptor is a heterodimer comprising the calcitonin receptor-like receptor (gene: Calcrl, protein: CLR) and receptor activity modifying protein 1 (gene: Ramp1, protein: RAMP1), which is expressed at higher levels in LEC than in blood endothelial cells.^303,304^ The CLR signaling pathway is critical for the development and maintenance of mouse and human vasculature.^305,306^ Additionally, CLR signaling plays a vital role in the lipid absorption of intestinal lymphatic vessels and the contractile function of lymphatic vessels in mice.^307,308^ Ramp1 gene deletion also results in restricted growth and functionality of lymphatic vessels.^309–313^

In 2019, Johnson et al. reported the use of CGRP antibodies in the treatment of trigeminal neuralgia.^314^ Research by Mikhailov et al. published in 2022 revealed that mLVs are involved in the pathogenesis of migraines, and this involvement is associated with localized inflammation within mLVs.^315^ Nelson-Maney NP et al., in 2024, discovered that the CGRP signaling pathway is a key mediator of LEC injury and dysfunction in a migraine model. In this study, CGRP gene knockout mice presented reduced migraine symptoms, and the injection of CGRP into the cisterna magna led to reorganization of LEC junctions and gap spaces and decreased drainage function.^316^ These findings suggest that the CGRP signaling pathway may be a key molecule that mediates LEC injury (Fig. 7e). However, validation of this molecule has thus far only occurred in migraine models, and further validation in additional models is necessary in the future. CGRP represents a potential crucial target in the pathogenesis of LEC injury.

Within the CGRP-CLR signaling pathway, activation of the CLR receptor stimulates LEC proliferation by activating the downstream MAPK/ERK and pAKT signaling pathways.^317^ The activation of CLR induces the reorganization of adherens and tight junction proteins in LECs, thereby reducing the permeability of these junctions. However, the recent identification of LYVE-1^+^ cells within the structure of arachnoid granulations (AGs),^87^ as well as the discovery of ACE structures,^79^ suggest that CSF can enter mLVs through multiple routes. With advancing insights into mLVs, scientists have discovered that mLVs in different locations exhibit unique characteristics, with those at the base of the skull seemingly playing a more pivotal role in CSF drainage. Furthermore, CSF absorption cannot be solely verified through changes in the permeability of mLVs, particularly in light of studies such as those by Zhang et al., which identified lymphatic emboli within mLVs following IVH. In addition, a 2019 article reported that the expression levels of endothelial junction proteins, such as VE-cadherin, decrease in aged mice,^78^ whereas an increase in VE-cadherin expression mediated by the CGRP-CLR signaling pathway has been observed in migraine models. The authors also suggest considering different types of endothelial cell junctions rather than solely comparing VE-cadherin levels. Therefore, a deeper investigation of the mechanism of action of the CGRP-CLR signaling pathway in intracranial mLVs is needed, especially considering the distinct roles it may play in different models.

### CcO signaling pathway

Recent studies have shown that photobiomodulation (PBM) has achieved promising outcomes in the treatment of brain injuries.^318–320^ PBM regulates lymphatic vessel function by modulating vasoactive responses.^321–323^ Research indicates that the mechanism of transcranial photobiomodulation (tPBM) treatment involves targeting brain metabolism, inflammation, oxidative stress, and neurogenesis.^324^ Furthermore, low-level laser therapy in mice with TBI has been shown to increase brain-derived neurotrophic factor (BDNF) levels and synaptogenesis.^325^ Another study demonstrated that transcranial laser stimulation can improve cerebral oxygenation in humans.^326^ The widely accepted view is that PBM activates photoreceptors such as cytochrome c oxidase (CcO), which produces ATP and promotes mitochondrial respiration, thereby improving mitochondrial activity.^324–326^ A 2019 study by Zinchenko et al. suggested that PBM may be a promising therapeutic target to prevent or delay AD.^327^ In 2024, Wang et al. demonstrated that NIR light enhances mitochondrial respiration in LECs through photoreceptor CcO, leading to the repair of lymphatic endothelial junctions and normalization of the alignment of mLV drainage patterns. This process facilitates the clearance of waste macromolecules such as Aβ, ultimately ameliorating the neurodegenerative process in mice.^328^

In summary, PBM activates CcO which in turn enhances the efficiency of mitochondrial respiration and ATP synthesis in LECs, thereby improving their viability and facilitating functional repair.^329^ It also promotes the upregulation of Cdc42S,^330^ leading to the repair of LEC endothelium. Furthermore,some studies suggest that the activation of CcO generates NO, which stimulates the production of ATP^331^ and is associated with reactive oxygen species signaling pathways^332^ (Fig. 7f). However, the mechanisms underlying these effects require further investigation.

### NO signaling pathway

Despite its simple structure, NO is a multifunctional molecule that plays an extensive role in various systems, including the cardiovascular system, CNS, reproductive system, endocrine system, respiratory system, and digestive system.^333^ NO is involved in a myriad of cellular signaling pathways^334,335^ and affects numerous cellular processes,^336^ with complex regulatory mechanisms.^337,338^ Studies have shown that hemoglobin can stimulate NO production following hemorrhagic stroke.^339–342^ NO induces vasodilation by activating soluble guanylate cyclase, which leads to the formation of cGMP and the activation of protein kinase G. This cascade opens calcium-activated potassium channels, increases Ca2^+^ reuptake, and inhibits myosin light-chain kinase, ultimately resulting in the relaxation of lymphatic vessels.^343^ Research by Li et al. in 2023 indicated that PBM therapy dilates mLVs and increases the production of NO in LECs, which is associated with increased contraction of lymphatic vessels^52^ (Fig. 7g).

In summary, the NO signaling pathway is a potential molecular mechanism regulating the contractility of mLVs. Interestingly, studies by Wu et al.^128^ and the author^46^ suggested that elevated levels of NO may be associated with lymphatic dysfunction, which seems to contradict the findings of Li et al. ^52^ In the hypothesis of the author’s study, lymph thrombi formed after IVH may lead to the dilation of basal mLVs and an increase in NO levels.^46^ However, alterations in NO levels should not be simplistically regarded as indicators of damage since the regulatory mechanism of NO is complex, and NO levels are likely subject to dynamic changes associated with its role in regulating lymphatic contractions.^344^ Therefore, the regulatory mechanism of NO in mLVs merits further investigation.

### CX3CR1 signaling pathway

CX3CR1 is commonly expressed in leukocyte populations^345,346^ but is typically expressed at lower levels in healthy vasculature and other tissues. Its ligand, CX3CL1 (also known as fractalkine), is broadly expressed in human neurons,^347^ epithelial cells,^348^ and macrophages^349^ and is infrequently found in peripheral blood cells.^350^ The CX3CL1/CX3CR1 axis mediates the recruitment of white blood cells and regulates the interactions between leukocytes and vascular endothelial cells, playing a role in the pathogenesis of atherosclerosis. CX3CR1 deficiency can ameliorate the severity of plaques in mouse models of atherosclerosis. Studies have implicated the overexpression of CX3CR1 in various pathological processes, including atherosclerosis,^351–354^ inflammatory damage,^354,355^ and angiogenesis disorders.^355^ Research by the author’s team in 2024 demonstrated that CX3CR1 is a key molecule in NET-induced LEC injury and meningeal lymph thrombosis, leading to mLV dysfunction, exacerbated hydrocephalus, and brain injury.^46^ CX3CR1 may be a crucial target for preventing post-IVH obstruction of meningeal lymphatic drainage^46^(Fig. 7h).

### Future directions in mLV injury signaling pathways

The formation of lymphatic vessels primarily encompasses four processes: proliferation, germination, migration, and the formation of vascular structures.^356^ While mLVs share some similarities with the developmental pathways of peripheral lymphatics, differences also exist. For example, during peripheral lymphatic development, the interactions of VEGFC and VEGFD with their receptor VEGFR3 serve as the main driving force.^231,357^ Additionally, the binding of VEGFA to VEGFR2 promotes the development of human lymphatics.^162^ Dimers formed by VEGFR2 and VEGFR3 facilitate LEC migration and lymphangiogenesis.^358,359^ However, current research on the CNS indicates that VEGFC is the primary regulator and that the absence of VEGFD does not impair the development of mLVs in mice.^75^ In 2017, a study by Zhong et al. identified VEGFR2 as a key molecule in corneal lymphangiogenesis^161^; However, a 2024 study by das Neves et al. indicates that blocking VEGFR2 does not impact the development and maturation of mLVs.^360^ Nonetheless, the regulatory role of the VEGFA signaling pathways in the development of central mLVs remains unclear.

In humans, mutations in the following 16 genes have been shown to cause various types of lymphatic diseases, most of which are involved in various aspects of lymphatic development^162^: VEGFR3,^361–364^ Foxc2,^261,262,365–370^ CCBE1,^371–376^ Gata2,^377–380^ Piezo1,^381^ Sox18,^382–384^ Fat4,^385–387^ ADAMTS3,^388^ FBXL7,^389^ GJC2,^390–392^ PTPN14,^393^ KIF11,^394^ ITGA9,^395–397^ REELIN,^398,399^ EPHB4,^400^ and CALCRL.^305^ Among these genes, VEGFR3, Foxc2, CCBE1, Gata2, and Piezo1 have been reported in mLV research. Moreover, molecules such as CX3CR1 have not been reported in studies of peripheral lymphatic injury; thus. further investigation into their potential roles in peripheral injury is merited. The author suggested that Sox18, Fat4, ADAMTS3, FBXL7, GJC2, PTPN14, KIF11, ITGA9, REELIN, EPHB4, and CALCRL could be potential targets for the investigation of mLV injury mechanisms (see Table 3 for details).

**Table 3:** Genetic associations with symptomatic lymphatic disorders and mLV research

Genes (symptomatic lymphatic disorders)	Disease	mLV Research	Reference
VEGFR3	Nonne–Milroy disease	Yes	^361–364^
Foxc2	Lymphedema–distichiasis (LD) syndrome	Yes	^261,262,365–370^
CCBE1	Hennekam lymphangiectasia–lymphedema syndrome type 1	Yes	^371–376^
Gata2	Emberger syndrome	Yes	^377–380^
Piezo1	Generalized lymphatic dysplasia (GLD)	Yes	^381^
SOX18	Hypotrichosis–lymphedema-telangiectasia (HLTS) syndrome		^382–384^
Fat4	Hennekam lymphangiectasia–lymphedema syndrome 2		^385–387^
ADAMTS3	Hennekam lymphangiectasia-lymphedema syndrome 3		^388^
FBXL7	Hennekam lymphangiectasia–lymphedema syndrome		^389^
GJC2	Late-onset autosomal dominant lymphedema		^390–392^
PTPN14	Choanal atresia and lymphedema		^393^
KIF11	MCLMR		^394^
ITGA9			^395–397^
REELIN			^398,399^
EPHB4			^400^
CALCRL	Hydrops fetalis with lymphatic dysplasia		^305^

The majority of injury molecules reported for mLVs are associated mostly with the VEGFC‒VEGFR3 axis or the maturation of lymphatic valves. Studies indicate that Akt, Yoda1, and ERK1/2 are involved in regulating the Foxc2 signaling pathway, whereas CGRP and ERK1/2 participate in regulating the VE-cadherin signaling pathway, and CCBE1 is involved in the regulation of the VEGFC-VEGFR3 signaling pathway. PBM regulation is concurrently involved in the activation of CcO and the production of ATP and NO. Given that the molecular pathways underlying the development and injury of mLVs remain unclear, we provisionally categorize the injury pathways according to the reported molecules (see Fig. 7), while the signaling pathways between these molecules are likely complex and interactive; consequently, further in-depth studies are warranted. Currently, research on the mechanisms of injury in diseased states has focused primarily on several aspects: ① injury to LECs, evaluated with (1) injury markers (VE-cadherin, TUNEL, CX3CR1), and (2) representative lymphatic endothelial activity indicators (Prox1 and LYVE-1, VEGF-C, Gata2). (3) Functional indicators (FOXC2 [lymphedema and lymphatic valve dysfunction] and NO [LEC contractile function]; and ② drainage obstruction caused by lymphatic thrombosis (lymphatic thrombosis due to NET-mediated fibrin deposition). Please refer to Table 4 for further details.

**Table 4:** Molecular mechanisms underlying mLV injury

Classification of mechanistic research	Indicator	Representative biological process	Reference
LEC injury marker	VE-cadherin	Disruption of LEC junctions	^46,47,49,78^
	CX3CR1	Exacerbation of the inflammatory response	^46^
	TUNEL	LEC injury	^46,51^
LEC activity marker	Prox1	Restricted proliferation of LECs	^62,75^
	LYVE-1	Restricted proliferation of LECs	^56,75,128^
	VEGF-C	Restricted proliferation of LECs	^56,75,128^
	Gata2	Restricted proliferation of LECs	^58^
mLV dysfunction marker	NO	Dysregulation of lymphatic vessel dilation function	^46,52,128^
	FOXC2	Lymphedema, lymphatic valve damage	^56,58,78,128^
Lymphatic coagulation	Fibrin	Lymphatic coagulation formation	^46^

## mLVs and CNS diseases

Studies indicate that mLVs are associated with neurodegenerative diseases, TBI, hemorrhagic stroke, ischemic stroke, infections, tumors, functional neurologic disorders, hepatic encephalopathy, and secondary hydrocephalus. The underlying mechanisms involve the transport of CSF, metabolic byproducts, and immune cells, in addition to interactions between the central and peripheral immune systems.

### mLVs and aging

Aging is an inevitable physiological process that occurs with advancing age; although it does not necessarily result in neurodegenerative diseases, it significantly increases the risk of such disorders. Recent studies have revealed a close link between aging and alterations in the glymphatic system, particularly how mLV dysfunction can facilitate the accumulation of toxic proteins in the brain, leading to the onset of various neurodegenerative diseases.^401,402^ This section discusses the changes in mLV during the aging process.

Specifically, the glymphatic system and mLV play crucial roles in maintaining cerebral metabolic balance and the clearance of toxic proteins. They facilitate the interchange of CSF and interstitial fluid^403,404^ and rapidly remove metabolic waste,^405–407^ thereby preserving homeostasis within the brain.^408^ Research indicates that mLVs play important roles in the clearance of metabolic byproducts^409^ during aging,^410–413^ cerebrovascular diseases,^414^ and sleep.^415^ The decrease in CSF outflow with age is attributed to reduced CSF production, changes in intracranial circulation, and impaired lymphatic drainage.^26,27,29–31,35^ For example, Ma et al. reported a significant decrease in CSF lymphatic drainage in aged mice compared with young mice in 2017, suggesting that the lymphatic system may represent a target for age-related neurological diseases.^23^ Furthermore, A study by Ahn et al. in 2019 reported that the integrity of mLVs and the drainage of CSF are compromised with increasing age.^78^ Aged mice exhibit characteristics of lymphatic edema in their mLVs.^78^ The underlying mechanism involves a more rapid deterioration of dorsal mLVs than of basal mLVs when VEGF-C signaling is lost. However, whether VEGF-C-VEGFR3 signaling is affected during aging and whether it can be targeted to counteract the observed age-related changes in mLV function remain unclear.^78,219^ Aging results in an abnormal distribution of type IV collagen in the basal mLVs of aged mice, and these vessels have fewer lymphatic valves than do those from young mice.^78^ Additionally, LECs in aged basal mLVs had 40.5% fewer zipper-type junctions and 1.6-fold more button-type junctions than those in young mice.^78^ Hence, the modulation of meningeal and cranial lymphatics is considered a potential therapeutic strategy for treating aging-related conditions.^413,416^ In summary, there is a significant correlation between aging and functional decline in the lymphatic system of brain, particularly in the structure and function of mLVs. This decline may increase the risk of progression of neurodegenerative diseases, suggesting the need for further research into the specific mechanisms by which aging affects mLVs, as well as the development of therapeutic strategies aimed at these changes.

### mLVs and neurodegenerative diseases

Neurodegenerative diseases, including AD and PD, are a group of disorders characterized by progressive neuronal death in the brain. The pathogenesis of these diseases involves complex mechanisms.^417–419^ These diseases share the common feature of abnormal accumulation of misfolded proteins in the brain, leading to neuronal degeneration and dysfunction. Recent studies have suggested that the onset of neurodegenerative diseases is closely linked to the dysfunction of mLV-mediated clearance of metabolic products.

AD, an age-related neurodegenerative condition, leads to personality alterations and marked cognitive impairments, including profound memory loss. The occurrence of AD is associated not only with the degeneration of the mLVs structure and function due to aging but also, as recent studies suggest, involves the depletion of PBMs,^420^ the mutation of the E4 variant of apolipoprotein E (APOE4), and a decline in functional hyperemia, also known as neurovascular coupling. PBMs in the mammalian brain play a vital role in the flow of CSF through lymphatic pathways (promotion of lymphatic clearance).^421,422^ Research by Da Mesquita et al. published in 2024 suggested that rejuvenating aged PBMs might hold promise in the treatment of age-associated neurodegenerative conditions, including AD.^423^ In transgenic mouse models of AD, the accumulation of Aβ in the brain with increasing age correlates with changes in the activation features of PBMs, including reduced expression of LYVE-1 and increased expression of major histocompatibility complex class II.^194,424^ With advancements in the study of meningeal lymphatics and the cranial lymphatic system, researchers have shown that while microglia play a role in phagocytosing metabolic products, PBMs regulate meningeal lymphatic-driven CSF flow,^425^ facilitating the drainage of metabolic products into the CSF and mLVs. The APOE4 is a major genetic risk factor for AD.^426–428^ Pericytes in the blood‒brain barrier secrete APOE4, and pericytes adjacent to mLVs may also secrete APOE4, influencing the structure and function of the meningeal lymphatics.^429^ Studies indicate that overexpression of APOE4 can lead to pericyte degeneration and result in cognitive impairment.^430^ APOE isoforms in the CSF can be distributed throughout the brain via lymphatic pathways in mice.^431^ APOE4 may exert some of its AD-related effects through mLVs,^184^ and its actions may result in early atrophy and functional deterioration of these vessels, leading to decreased CSF flow.^432^ In 2021, Mentis et al. proposed a conceptual framework in which APOE4 plays a novel role in the premature atrophy of mLVs (meningeal lymphatic sclerosis), leading to dysfunctional meningeal lymphatics (meningeal lymphedema) and consequently reducing the clearance of Aβ, other macromolecules, inflammatory mediators, and immune cells.^433,434^ Additionally, APOE4 is an independent risk factor for ischemic vascular diseases.^435^ However, further validation of this mechanism is needed. Neurovascular coupling is a phenomenon in which increased neural activity triggers an increase in local cerebral blood flow. In 2023, research by Holstein-Rønsbo et al. revealed that whisker stimulation augmented lymphatic influx and clearance in the somatosensory cortex of mice, increasing the CSF inflow speed surrounding the middle cerebral artery by 1.6 times.^406,407^ However, whether neurovascular coupling can regulate neurodegenerative pathology remains unclear. In a 2024 study by Zhou et al. of a rat model of white matter hyperintensity, evidence of impaired lymphangiogenesis and lymphatic drainage was obtained, followed by the activation of microglia and increased demyelination of white matter.^436^ This study provides insight into how the dysfunction of mLVs and the glymphatic pathway may exacerbate white matter damage, which is significantly correlated with AD. In 2024, Ye et al. discovered that borneol (BO) facilitates the formation of valves in mLVs and may prevent or repair damage induced by toxic Aβ42.^263^ In recent years, promising effects of PBM therapy in combating aging have been reported.^437,438^ In 2024, Shan et al. reported an increase in lymphatic vessel and meningeal coverage, as well as accelerated clearance of macromolecules in the brain, following the administration of a long-acting lyotropic liquid crystalline implant encapsulating cilostazol (an FDA-approved selective PDE-3 inhibitor) and donepezil hydrochloride (a commonly used symptomatic relief agent that inhibits acetylcholinesterase in individuals with AD) near the dCLNs of aged mice.^439^ Research by Semyachkina-Glushkovskaya et al. published in 2024 revealed that, compared with daytime PBM, nighttime PBM could be a more promising method for preventing AD.^440^ Furthermore, in 2024, Wang et al. identified the subcutaneous injection of nanomaterial drugs near CLNs as a means to deliver therapy centrally and ameliorate mLV function.^441^ Recent preliminary successes in treating dementia with dCLN venous anastomosis^152,153^ have opened new research directions in the treatment of aging.

PD represents another neurological disorder induced by aging,^442^ afflicting over six million individuals worldwide^443^ and significantly endangering human health.^442,444^ The clinical treatment of PD primarily includes pharmacological interventions administered orally or intravenously.^443,445–448^ However, the blood‒brain barrier substantially limits the efficiency of drug entry into the brain,^449,450^ and current therapeutic practices also face challenges such as improper dosing.^451^ Although intranasal administration has achieved considerable advancements,^452,453^ enzymatic degradation within the nasal and mucosal endothelial barriers may diminish drug activity and reduce absorption, respectively.^454^ Therefore, exploring effective drug delivery pathways is crucial for enhancing the therapeutic outcomes of PD. In 2023, Liu et al. introduced a natural killer cell membrane biomimetic nanocomplex administered via the mLV route to improve the therapeutic efficacy for PD. This study highlights the promising potential of the mLV route for targeted drug delivery to the brain, providing substantial hope for the treatment of neurodegenerative diseases.^455^ However, the critical question remains unanswered: can mLV-directed therapy effectively inhibit or reverse neurodegenerative processes in humans? Furthermore, what are the long-term consequences of such interventions? How can we tailor them most effectively to individual patients? Addressing these gaps will pave the way for innovative and clinically viable treatment approaches for neurodegenerative diseases.

### mLVs and TBI

TBI is a major global health concern^456^ and involves complex mechanisms of injury.^457,458^ Recent studies suggest that post-TBI injury mechanisms involve meningeal lymphatics^459,460^ and mLVs.^461^ The development of cerebral edema following TBI is associated with drainage through mLVs and meningeal lymphatics.^460^

Cerebral edema is associated with the incidence and mortality rate following TBI. A study published by Bolte et al. in 2020 indicated that mild TBI results in severe defects in mLV drainage, which can occur within hours and persist for at least one month postinjury. Restoration of mLV drainage can increase TBI-induced neuroglial proliferation.^41^ Research by Hussain et al. published in 2023 investigated the relationship between brain edema post-TBI and the lymphatic system, revealing the involvement of the mLV system in the development of brain edema following TBI and in the drainage of postinjury metabolites (cell debris, etc.).^45^ The findings of a study conducted by Liao et al. in 2023 suggested that ketorolac, 9-cisRA, and VEGF-C can increase the integrity of the mLV structure and promote lymphatic proliferation by increasing the expression of lymphatic-specific proteins. This improvement in mLV function facilitates CSF drainage and brain edema absorption, reduces the immune response of the nervous system, decreases reactive oxygen species formation, and consequently leads to better outcomes post-TBI.^42^ A 2023 study by Li et al. indicated that exogenous IL-33 improved motor and cognitive functions in mice with TBI by enhancing acute mLV drainage.^43^ Recently, Hussain et al. identified a novel type of brain edema, termed glymphatic-stagnated brain edema, resulting from compromised glymphatic and mLV drainage due to noradrenergic activation in TBI.^44^

In conclusion, the mLV system plays a role in disease progression following TBI, and promoting mLV drainage can facilitate the resolution of edema after TBI. The regulation of mLVs involves central immunity and communication between central and peripheral immunity. While current research has reported the role of meningeal lymphatics after TBI, researchers have not clearly determined whether the simultaneous modulation of meningeal and cortical lymphatics can more effectively ameliorate damage following TBI. The mLV system represents a potential therapeutic target for TBI, yet additional fundamental research efforts are needed in the field.

### mLVs and hemorrhagic stroke

In recent years, increasing scholarly attention has been given to the role of mLVs in hemorrhagic stroke, encompassing research on diseases such as ICH, IVH, SAH, and epidural hematoma.

#### mLVs and ICH

ICH is a severe neurological disorder^462,463^ with a high mortality rate and substantial risk of serious complications.^464–466^ Its postinjury mechanisms are complex.^467^ In 2022, Tsai et al. conducted a study on ICH model mice in which the research team evaluated the drainage function of the mLV system utilizing CSF tracers and PKH-26-labeled RBCs. Post-ICH, RBCs can drain through the mLV system to the CLNs, and impaired mLV function can exacerbate brain damage by increasing iron deposits around the ICH site, increasing the number of residual RBCs and promoting neuronal apoptosis, thus worsening brain injury. These findings indicate the involvement of mLVs in the pathogenesis of brain damage following ICH.^54^ Additionally, the team’s results suggested that the generation of mLVs and enhancement of lymphatic drainage occur in the late stages of ICH, with early enhancement of mLV function identified as beneficial for ICH recovery. In summary, mLVs play a role in pathological progression following ICH via mechanisms involving the drainage of extravasated RBCs and their metabolic byproducts (iron deposits) by mLVs. Improving mLV function post-ICH represents a potential therapeutic target. Additionally, the metabolic byproducts released by RBCs following ICH can trigger the activation of the central immune system. How to control the inflammatory storm through mLVs or mitigate its central effects, as well as its peripheral consequences or influence on the central system, is an issue worthy of investigation.

#### mLVs and IVH

Posthemorrhagic hydrocephalus occurs in up to 50% of patients after ICH,^468^ representing a severe complication of ICH.^469–471^ Currently, surgical intervention and thrombolytic pharmacotherapy are the main modalities for treating posthemorrhagic hydrocephalus. However, invasive CSF shunting procedures carry the risk of serious complications.^472^ The outcomes of the highly anticipated IVH Thrombolysis trial (CLEAR III), a phase III clinical study for the treatment of IVH, have been disappointing.^473^ The pathogenetic mechanisms of early hydrocephalus following ICH include impediments to CSF flow,^474,475^ aberrant CSF production by the choroid plexus,^469,470^ and fibrosis of AGs.^476,477^ However, recent studies have identified the perineural spaces around cranial nerves,^21,22^ mLVs,^21^ and the choroid plexus itself^19,20^ as pathways for CSF egress from the cranium. Consequently, the study of mLVs in the context of ICH has become a focal point of current research. A study by Li et al. in 2024 demonstrated that following IVH, mLVs serve as a pathway for the clearance of RBCs from the ventricular system of the brain in male humans, as well as in adult and newborn rodents. Furthermore, these mLVs present a target for noninvasive transcranial NIR therapy.^52^ In 2024, Zhang et al. studied IVH model mice and reported that, following IVH, significant accumulation of neutrophils, neutrophil extracellular traps (NETs), and fibrin occurs within mLVs. These NETs within mLVs induce acute damage to LECs, triggering lymphatic thrombosis. Furthermore, NETs promote the upregulation of CX3CR1 expression on LECs, leading to increased neutrophil aggregation and increased NET formation, further exacerbating damage to the mLV system. These findings provide new insights into the pathophysiological mechanisms underlying secondary hydrocephalus following hemorrhagic stroke and pave the way for novel research directions for future therapeutic strategies.^46^ In conclusion, mLVs act as a pathway for the removal of RBCs from the ventricular system following IVH. Furthermore, NETs induced by IVH result in damage to and dysfunction of the lymphatic endothelium, leading to lymphatic thrombosis, mLV involvement in secondary hydrocephalus and brain damage post-IVH. CX3CR1 has emerged as a key molecule in NET-mediated injury to LECs.

#### mLVs and SAH

SAH represents a serious health-threatening condition.^478–480^ The first paper addressing cerebral lymphatic drainage in the process of cerebral injury following subarachnoid hemorrhage was reported by Sun et al. ^481^ These authors reported that blocking cerebral lymphatic drainage exacerbates cerebral oxidative injury in rats with subarachnoid hemorrhage. A subsequent study was by Pu et al. reported persistent malfunction of glymphatic and meningeal lymphatic drainage in a mouse model of SAH.^170^ In 2020, research by Chen et al. revealed a significant increase in the accumulation of RBCs in dCLNs and mLVs following SAH. The findings of this work indicated that mLVs drain extravasated RBCs from the CSF into the dCLNs after SAH.^174^ In 2023, Wang et al. reported that the administration of dobutamine post-SAH increases the clearance of RBCs and their degradation products through brain mLVs, thereby alleviating early neurological deficits post-SAH.^53^ A study conducted in 2023 by Yang et al. on a mouse model of SAH revealed that this condition leads to damage to LECs. Further investigation revealed that after SAH, thrombospondin-1 (THBS1)-CD47 signaling via STAT3/Bcl-2 regulates endothelial cell apoptosis in mLVs.^51^ In conclusion, mLVs are involved in pathological progression following SAH, with mechanisms involving the drainage of extravasated RBCs and other metabolic byproducts from the CSF by mLVs. Enhancing the function of mLVs after SAH represents a potential therapeutic target. However, current reports present some controversies. Chen et al.^174^ reported an increase in SAH-related mLV drainage following SAH, a viewpoint that contradicts the prevailing opinions in the field, necessitating further research to elucidate the underlying mechanisms.

#### mLVs and SDH

In 2020, Liu et al. reported that RBCs drain through mLVs to CLNs, whereas SDH leads to reduced expression of LYVE-1, FOXC2, and VEGF-C in the meninges.^56^ In 2024, through a study on SDH model rats, Yuan et al. discovered that RBCs are drained into CLNs via mLVs. SDH induces ERK1/2 dephosphorylation in mLV endothelial cells, resulting in primary mLV destruction and impaired drainage through mLVs. Atorvastatin alleviates post-SDH damage by strengthening the basolateral connections of mLV endothelial cells.^47^ In 2024, Chen et al. induced SDH in Sprague‒Dawley rats and then treated the rats with vitamin D; a reduction in the SDH volume and improvement in drainage to the CLNs were observed. These improvements were associated with the restoration of LYVE-1, PROX1, FOXC2, and VE-cadherin expression. Additionally, vitamin D alleviated neuroinflammation in brain tissue, protecting the structure of mLVs.^49^ The findings of a prospective study published in 2024 by Zhang et al. on chronic SDH indicated a correlation between the impaired drainage function of mLVs and the risk of recurrence (*p* < 0.05). Through noninvasive MRI, this study revealed dysfunction in mLV drainage after CSDH, with mLV drainage function being an independent predictor of CSDH recurrence.^50^ In summary, mLVs play a role in the disease progression of SDH, suggesting that improving mLV function could be a potential target for SDH treatment. Vitamin D improves LEC function and junctions within the endothelium via the ERK1/2 signaling pathway, but further research is needed to clarify its molecular pathways. In particular, clinical studies have provided additional support for findings currently observed in basic research. The authors believe that with more in-depth studies, the role of mLVs in SDH will be further revealed.

### mLVs and ischemic stroke

In recent years, mLVs have emerged as a research hotspot or potential intervention method for ischemic stroke.^482–485^ In 2022, Bai et al. subjected MCAO model rats to cranial bone transport and reported improved neurological outcomes, with mechanisms linked to increased mLV drainage with cranial bone transport.^55^ In 2024, Yang et al. utilized AAV-VEGF-C to promote mLV restoration, thereby ameliorating neuroinflammation and improving the MCAO prognosis.^48^ The findings of this study provide a theoretical basis for the role of mLVs in central immune cell infiltration and peripheral immune cell activation. In conclusion, mLVs are involved in the progression of ischemic stroke, suggesting that improving mLV function could be a potential target for treating poststroke hemorrhage. Following a stroke, mLVs transport central products and immune cells to the periphery, amplifying systemic inflammation. Current reports indicate that blocking the CLN can attenuate intracranial inflammation in MCAO, although this viewpoint remains controversial. Therefore, further research is necessary.

### mLVs and infection

Recent research suggests that mLVs are closely associated with the onset and progression of central infectious diseases, with mechanisms involving the central immunity of mLVs and interactions between mLVs and peripheral immunity. Currently reported mLV-related infectious diseases include bacterial infections, viral infections, and parasitic infections.

#### mLVs and bacterial infections

Sepsis-associated encephalopathy (SAE) is an acute form of brain dysfunction caused by sepsis. In a study by Huang et al. in 2023, a model of SAE induced by the intraperitoneal injection of LPS was established, and meningeal resident immune cells and mLVs were examined. Systemic exposure to LPS induced the recruitment, extravasation, and aggregation of neutrophils around meningeal blood vessels. Additionally, the shape and position of meningeal resident macrophages are altered after LPS injection, downregulating the expression of major histocompatibility complex class II, thus improving the ability of mLVs to promote inflammation recovery.^60^ In 2024, Dong et al. investigated mice with lipopolysaccharide (LPS)-induced SAE and reported that AAV-VEGF-C reduced microglial cell activation and neuroinflammation, leading to an amelioration of cognitive impairment. An increase in mLV function also decreases the expression of sepsis-induced disease-related genes in aged mice.^59^ LM infection is a serious condition that can affect multiple organs and lead to fatal outcomes. According to a 2024 study by Feng et al., LM infection suppresses the expression of genes crucial for lymphatic vessel development, such as Gata2 and Foxc2, thereby impairing the drainage function of mLVs in mice.^58^ Bacterial infectious diseases associated with mLVs include central bacterial infections and peripheral bacterial infections, indicating that mLVs are pivotal in mediating interactions between central and peripheral immunity. mLVs play a role in the pathophysiology of CNS bacterial infections via mechanisms such as neutrophil recruitment and microglial cell activation, which may be associated with the downregulation of Gata2 and Foxc2 gene expression. However, whether bacterial infections other than sepsis and LM affect mLVs is currently unclear.

#### mLVs and CNS parasitic infections

In 2024, Kovacs et al. conducted a study on CSF drainage in mice infected with *Toxoplasma gondii* and reported that treatment with VEGF-C increased lymphatic outflow in infected mice, although this effect did not lead to increased clearance of edema fluid in the brain.^57^ Notably, the study by Kovacs et al., which utilized VEGFC to modulate mLV dysfunction caused by *Toxoplasma gondii*, failed to ameliorate cerebral edema, a phenomenon that warrants further consideration. However, whether concurrent modulation of mLVs and the glymphatic system (e.g., enhancing the activity of PBMs) can improve post-Toxoplasma infection-induced cerebral edema currently remains unclear.

#### mLVs and CNS viral infections

A study by Li et al. in 2022 investigated the impact of neurotropic viruses within the CNS of mice—specifically, Zika virus, Japanese encephalitis virus, rabies virus, herpes simplex virus I, and vesicular stomatitis virus—on the proliferation of intracranial mLVs. mLVs, which serve as drainage pathways for viruses from the CNS to dCLNs, exhibit impaired function due to these viral infections. Research has revealed that ligating lymphatics or photodynamic ablation of dorsal mLVs exacerbates neural damage and increases mortality rates following viral infections. Conversely, pretreatment with VEGF-C enhances mLV function, effectively mitigating the adverse effects of viral infections. These findings underscore the role of functional mLVs in the viral clearance process and their critical role as a key pathway for viral drainage from the CNS to dCLNs.^62^ Subsequently, Lemprière et al. commented on the aforementioned research, highlighting that functionally enhanced mLVs can improve the outcomes of CNS infections, suggesting that this mechanism could be utilized for therapeutic interventions.^61^ Does the proliferation of mLVs signify enhanced function? Research by Li et al. in 2022 demonstrated that while dorsal mLVs proliferate in mice with viral meningitis, their drainage function is impaired. The authors also discussed how the diverse functionalities of newly formed mLVs might be determined by varying disease conditions or localities.^62^ Additionally, their study on the IVH model indicated that lymphatic thrombosis within mLVs could be the cause of impaired mLV drainage.^46^ Moreover, basal mLVs not only serve as crucial hubs for CSF drainage but also act as downstream drainage areas for dorsal mLVs. When basal mLVs experience functional impairments such as lymphatic thrombosis, this change could lead to a compensatory increase in the number of dorsal mLVs. In conclusion, mLVs play a significant role in the pathogenesis of viral meningitis, and promoting the effective proliferation of mLVs can facilitate infection recovery by regulating mLV function. Infections previously reported to be associated with mLVs include bacterial, viral, and parasitic infections. However, the relationship between central fungal infections and mLVs remains unclear.

### mLVs and tumors

Traditional views suggest that owing to the lack of lymphatic drainage in the intracranial space, the immune surveillance capacity of the CNS against pathogens and tumors is relatively limited. However, recent studies on the characteristics of the mLV network revealed a previously underappreciated mechanism that may trigger immune responses against antigens expressed within the brain.^16,17,132^ A study by Ma et al. in 2019 revealed reduced CSF lymphatic outflow in patients with glioma.^486^ In 2020, Song et al. utilized the VEGF-C signaling pathway to modulate the mLV system in a glioblastoma mouse model to improve the immune response to brain tumors. The results revealed the ability of VEGF-C to increase tumor immune surveillance.^63^ In 2020, Hu et al. reported extensive remodeling of dorsal mLVs in mice with intracranial gliomas or metastatic melanomas. Tumor-bearing mice overexpressing VEGF-C exhibited an increased response to anti-PD-1/CTLA-4 combination therapy, suggesting that VEGF-C increases the efficacy of anti-PD-1/CTLA-4 therapy through the CCL21/CCR7 pathway. This research underscores the crucial role of mLVs in generating effective immune responses against brain tumors.^64^ As a frontline treatment, radiotherapy (RT) can modulate the immune microenvironment of glioblastomas, but whether the mLV–CLN network regulates this process or impacts the efficacy of RT remains unclear. In 2022, a study by Zhou et al. indicated that functional impairment of mLVs compromised DC trafficking and CD8+ T-cell activation post-RT in tumor-bearing mice, whereas VEGF-C overexpression in tumors with expanded mLVs resulted in high sensitivity to RT. Mechanistically, VEGF-C-driven immunomodulation triggered by RT was attributed to CCL21-dependent DC trafficking and CD8^+^ T-cell activation, highlighting the significant role of the mLV–CLN network in RT-induced antitumor immunity and emphasizing the potential of VEGF-C mRNA in brain tumor therapy.^65,66^ Wang et al. (2023) demonstrated that long-term decreased drainage of mLVs is a risk factor for tumor progression.^67^ In summary, mLVs contribute to the progression of brain tumors through mechanisms such as tumor immune surveillance, and further exploration is warranted. mLVs may play crucial roles in tumor immunity, highlighting the need for in-depth research into the mechanisms of action of dorsal mLVs in tumor immunology. Given that mLVs can serve as delivery vehicles for chemotherapeutic agents, mLVs represent new potential targets for the treatment of brain tumors.^66^ The authors suggest that drug delivery through the CLN and nasal routes may be a potential hotspot for modulating mLVs in the treatment of CNS tumors.

### mLVs and migraine and epilepsy

In 2024, Peng et al. conducted a study in which MRI was used to assess the drainage of brain lymphatic vessels adjacent to the sagittal sinus and mLVs alongside the transverse sinus in patients with chronic migraine compared with healthy controls. This study revealed functional impairments in the mLVs of migraine patients, suggesting that mLV drainage may play a role in the pathogenesis of migraine.^68^ Additionally, recent scholarly research has linked drainage dysfunction in mLVs to the pathogenesis of epilepsy.^69^ Taken together, these findings indicate that mLVs are involved in the pathophysiological processes of nonorganic CNS functional diseases, including migraine and epilepsy. Current research indicates that Piezo1 is associated with the regulation of ICP and blood pressure. However, whether the involvement of mLVs in the pathogenesis of organic CNS functional diseases is related to Piezo1 remains unclear.

### mLVs and hepatic encephalopathy

Hepatic encephalopathy (HE) is a severe neurological complication in patients with liver cirrhosis, and understanding its pathophysiology is crucial for developing effective treatments. In 2024, Hsu et al. conducted a study that provided significant insights into the role of mLVs in HE. Using AAV8-VEGF-C to enhance mLV function in a mouse model, they reported a marked improvement in cortical microglial activation and neuroinflammation, as well as increased drainage from the meninges and LNs.^487^ A study by Shu et al. in 2024 revealed that patients with metabolic dysfunction-associated fatty liver disease may experience glymphatic dysfunction prior to the onset of liver cirrhosis.^179^These studies collectively suggest that peripheral diseases can also affect the drainage of mLVs, warranting further investigation into whether this process is related to the interaction between peripheral immunity and the CNS. These findings suggest that impaired mLV drainage may contribute to the pathogenesis of HE, highlighting the potential of targeting mLVs to improve outcomes in patients with this condition.

### mLVs and secondary hydrocephalus

Impaired CSF circulation is a serious complication following ICH or IVH. In a pivotal study conducted in 2024, Zhang et al. utilized an autologous blood injection method to establish a mouse model of IVH and discovered extensive infiltration of neutrophils, fibrin, and NETs within mLVs post-IVH. The degradation of NETs is associated with a reduction in hydrocephalus and brain injury, a process that is linked to the activation of LECs and increased CSF drainage. This study revealed that following IVH, NETs activate the LEC membrane protein CX3CR1, which has been identified as a critical molecule in NET-induced LEC injury and mLV thrombosis. This activation leads to mLV dysfunction, exacerbating hydrocephalus and brain injury.^46^ In conclusion, these findings underscore the importance of mLVs in the pathophysiology of impaired CSF circulation after ICH or IVH, with implications for potential therapeutic interventions targeting mLV function.

In summary, recent studies have linked mLVs to a range of diseases, including neurodegenerative diseases,^23,25–40^ TBI,^41–45^ hemorrhagic stroke^46,47,49,50,52–54,56,174^ ischemic stroke,^48,55^ CNS infections,^57–62^ brain tumors,^63–67^ brain diseases caused by systemic diseases,^487^ functional neurological disorders,^68,69^ and secondary hydrocephalus (Table 5).^46^

**Table 5:** CNS diseases associated with mLVs

Disease classification	Disease name	Mechanisms of mLVs	Reference
Neurodegenerative diseases	Alzheimer’s disease	Drainage of aging astrocytes, cellular debris, tau protein and Aβ protein	^23,28,167^
	Parkinson’s disease	Drainage of alpha-synuclein	^37^
	Amyotrophic lateral sclerosis	TAR DNA-binding protein 43 and glutamate	^33^
Trauma	Traumatic brain injury	Drainage of RBCs mediates immune-related neuroinflammation	^41–45^
Hemorrhagic stroke	Intracerebral hemorrhage	Drainage of RBCs mediates immune-related neuroinflammation	^54^
	Subarachnoid hemorrhage	Drainage of RBCs mediates immune-related neuroinflammation	^51,53,170,174,481^
	Intraventricular hemorrhage	Drainage of RBCs mediates immune-related neuroinflammation	^46,52^
	Subdural hematoma	Drainage of RBCs mediates immune-related neuroinflammation	^47,49,50,56^
Ischemic stroke	Ischemic stroke	Drainage of metabolic byproducts and interstitial fluid to reduce edema	^48,55^
Infection	CNS bacterial infections	Mediates immune-related neuroinflammation	^58,60^
	CNS virus infections	Mediates immune-related neuroinflammation	^61,62^
	CNS parasitic infections	Drainage of CSF	^57^
Tumor	Tumor	Mediates immune-related neuroinflammation	^63–67^
Functional neurological disorders	Migraine	Drainage of CSF	^68^
	Epilepsy	Drainage of CSF and interstitial fluid	^69^
Brain disease caused by systemic diseases	Hepatic encephalopathy	Mediates immune-related neuroinflammation	^487^
Hydrocephalus	Secondary hydrocephalus	Drainage of CSF	^46^

## Potential regulatory mechanisms of mLVs

The regulation of mLVs can be classified on the basis of the site of modulation into the regulation of mLVs and cerical LVs. Pathways can be divided according to their mechanism of control into pharmacological methods and physical methods of regulation. Pharmacological regulation comprises agents such as VEGFC, BO, Yoda 1, dopexamine, vitamin D, yuanzhi powder (YZP) and DNAse I. Physical methods for regulating mLVs include NIR, repetitive transcranial magnetic stimulation (rTMS), acoustic regulation, and electrical stimulation, among others.The regulation of cerical LVs can be categorized into two approaches: pharmacological control and physiological modulation.

### Pharmacological modulation of intracranial mLVs

Pharmacological regulation of mLVs includes agents such as VEGFC, DNAse I, dobutamine, BO, Yoda 1, YZP, and vitamin D, among others.

#### VEGF-C regulation of mLVs

The therapeutic potential of VEGF-C in modulating mLVs has garnered significant attention in recent years. In 2018, Wen et al. demonstrated that VEGF-C injection in AD model mice promoted the proliferation of mLV endothelial cells and facilitated the drainage of the Aβ protein to CLNs. This finding,^28^ highlights the role of VEGF-C in increasing the clearance of neurotoxic proteins. Furthermore, In 2024, Boisserand et al. investigated the effects of VEGF-C overexpression on CSF drainage and outcomes following ischemic stroke in mice. Their study, cited as a reference, revealed that AAV-mVEGF-C pretreatment reduced brain damage caused by stroke and improved motor performance during the subacute phase. This improvement was associated with mitigated microglial-mediated inflammation and increased BDNF signaling within brain cells.^250^ In the same year, Da Mesquita et al. published a groundbreaking study, as referenced in,^39^ which showed that VEGF-C treatment could enhance the drainage of the Aβ protein through mLVs, providing further evidence of its therapeutic efficacy. Additionally, in 2022, Li et al. reported that VEGF-C pretreatment in mice with intracranial viral infections could enhance mLV function and effectively alleviate the negative effects of viral infection, as noted previously.^62^

In summary, VEGF-C has emerged as a promising therapeutic agent for a variety of animal disease models, including AD,^167^ stroke, and viral encephalitis. Taken together, these findings underscore the potential of VEGF-C to modulate mLVs, providing a novel approach for treating neurological diseases by increasing waste clearance and reducing inflammation. As research continues to evolve, the modulation of intracranial mLVs by VEGF-C has become a compelling avenue for therapeutic intervention.

#### DNase I regulation of mLVs after IVH

Recent studies have indicated that NETs can induce fibrin deposition in the lungs and regional LNs of coronavirus disease 2019 patients.^488^ Lymphatic thrombosis has also been reported in a variety of diseases and conditions, including cancer, infections, amyloidosis, and poor lymph node clearance.^489^ Zhang et al. discovered the presence of lymphatic thrombi within mLVs following IVH in a mouse model of IVH. They reported that the degradation of NETs during the acute phase of IVH could ameliorate hydrocephalus and neurological deficits. The underlying mechanism involves a cascade of damage responses induced by elevated CX3CR1 expression after IVH, leading to lymphatic thrombosis and drainage embolism caused by NET-mediated fibrin deposition. The formation of lymphatic thrombi within mLVs further exacerbates local brain injury.^46^ This study demonstrated that DNase I can degrade NETs within the mLV system, thereby reducing the formation of lymphatic thrombi and restoring the function of mLV endothelial cells. These findings provide a reliable basis for the regulation of mLVs by DNase I following hemorrhagic stroke.

In summary, the role of NETs in contributing to lymphatic thrombosis and subsequent tissue damage is increasingly recognized across various pathological conditions. The work by Zhang et al. highlights the therapeutic potential of DNase I in mitigating these effects by targeting NETs within the mLV system post-IVH. This research not only elucidates the pathophysiology of IVH but also opens new avenues for the treatment of hemorrhagic stroke through the modulation of mLVs.

#### Dobutamine modulation of mLVs

Dobutamine, a potent β1-adrenergic receptor agonist, is known to increase cardiac output, thereby improving cerebral blood perfusion and arterial pulsation. Recent findings reported by Wang et al. in 2023 elucidated the role of dobutamine in the context of SAH. The findings of their study suggested that the administration of dobutamine post-SAH facilitates the clearance of RBCs and their degradation products through mLVs. This process, in turn, alleviates early neurological deficits following SAH. In essence, the modulation of intracranial mLVs by dobutamine represents a novel therapeutic approach for managing the acute phase of SAH, with the potential to improve patient outcomes by increasing the clearance of hemorrhagic debris from the brain.^53^

#### Borneol (BO) modulation of mLVs

The therapeutic potential of BO, a traditional compound with a history of medicinal use, in neurological disorders has garnered increasing interest.^490^ This substance has been shown to exhibit neuroprotective, anti-inflammatory, and antiepileptic properties, primarily due to its ability to rapidly cross the blood‒brain barrier and accumulate in the brain at high concentrations.^491,492^ This ability has been linked to its applications in treating stroke, AD, and epilepsy. Furthermore, BO is known to increase local microcirculation by increasing the expression of vascular endothelial growth factor, providing a theoretical foundation for its use in treating cerebral diseases. In 2023, Wu et al. conducted a pivotal study focusing on the regulation of mLVs to promote the clearance of Aβ from the brain, a mechanism of particular relevance to AD. Their research involved treating AD model mice with BO and showed that BO effectively improved the drainage function of mLVs, thereby significantly facilitating the clearance of Aβ. These findings suggest that BO has a promising future as a potential therapeutic agent for AD.^128^ Earlier, in 2016, Tambe et al. highlighted the antiepileptic potential of BO in an epilepsy model.^490^ Collectively, these studies underscore the multifaceted biological activities of BO and its potential as a therapeutic agent for brain disorders. Ongoing research on the modulation of intracranial meningeal lymphatics by BO represents a promising avenue for the development of novel treatments for neurodegenerative diseases and other cerebral conditions.

#### Regulation of mLVs by Yoda1 (a Piezo1 agonist)

In the field of neurobiology, the year 2023 marked significant advancements in our understanding of mLVs and their role in cerebral homeostasis. Matrongolo et al. reported that in aged mice with reduced CSF drainage, treatment with Yoda1, a Piezo1 agonist, increased the function of the lymphatic network, lymphatic drainage, and CSF perfusion. These findings suggest that Yoda1 agonists could be a viable therapeutic option for ameliorating conditions characterized by elevated ICP and reduced CSF flow, such as craniosynostosis or aging, by restoring the mLV network and CSF perfusion.^493^ Similarly, Ma et al. utilized Twist1 mice, which are craniosynostosis model mice, to demonstrate that treatment with VEGF-C could promote the growth of mLVs and rescue the associated symptoms of increased ICP, impaired cerebral perfusion, and cognitive deficits. The findings of this study underscore the functional integration of the skull with the brain via mLVs, highlighting that mLVs are compromised in craniosynostosis and can be rejuvenated through VEGF-C-driven proliferation.^288^ In summary, the findings of these studies collectively illuminate the critical role of mLVs in maintaining cerebral health and provide promising therapeutic strategies for conditions involving impaired lymphatic drainage and CSF circulation. The potential of pharmacological agents such as Yoda1 and VEGF-C to modulate mLVs provides a foundation for future interventions aimed at restoring the delicate balance of ICP and fluid homeostasis, thereby safeguarding neurological function.

#### Yuanzhi powder modulation of mLVs

In 2024, Li et al. revealed the therapeutic potential of YZP in AD, demonstrating its ability to increase lymphatic drainage in a mouse model. This study highlights the dual effects of YZP: restoring AQP4 polarization to facilitate cerebral fluid balance and inhibiting reactive astrocyte proliferation to reduce neuroinflammation. Additionally, YZP expands the diameter and coverage of mLVs, promoting the clearance of brain waste.^494^ In summary, Li et al. reported that YZP is a promising candidate for AD treatment, as its targeted modulation of meningeal lymphatics potentially slows disease progression and preserves cognitive health.

#### Vitamin D regulation of mLVs

In 2024, Chen et al. revealed that vitamin D increases VE-cadherin expression in mLVs and increases the clearance of SDHs in mice.^49^ This pivotal study suggested that vitamin D could be a therapeutic agent for SDH, promoting mLV integrity and hematoma absorption. In summary, the research conducted by Chen et al. underscores the pivotal role of vitamin D in the therapeutic intervention of SDH by reinforcing the structural and functional integrity of mLVs.

### Physical modulation of mLVs

In recent years, physical methodologies have made certain advances in the research of modulating mLVs. For example, PBM has shown preliminary therapeutic effects on the regulation of AD. This section elaborates on the latest methods for modulating mLVs by combining the most recent research reports, with the aim of advancing progress in this field of study.

#### Near-infrared modulation of mLVs

PBM research, particularly that involving light in the NIR region,^495^ has been substantial, especially regarding the modulation of mLVs. Studies by Tao et al. have shown that 1070-nanometer NIR light can increase the phagocytosis of Aβ by microglia, improving cognitive functions in AD model mice.^496^ Additionally, Hamblin et al. reported that light ranging from 600 to 1100 nanometers can aid in wound healing, tissue protection, mitochondrial function enhancement, and blood circulation, reduce swelling, oxidative stress, and inflammation, and prevent cell death.^319^ In support of the versatility of PBM, Baxter et al. reported that PBM can effectively alleviate symptoms of lymphedema associated with breast cancer.^497^ Semyachkina-Glushkovskaya et al. reported that PBM at 1267 nm could be a novel strategy for preventing neurological diseases by enhancing the drainage and clearance functions of mLVs.^321^ In line with these findings, Li et al. demonstrated that the administration of 1267 nanometers of NIR light at a dose of 9 joules/square centimeter can modulate the contractility of mLVs through an NO-mediated mechanism, promoting lymphatic drainage and clearance in neonatal rats and expediting the removal of RBCs post-IVH.^52^ In 2023, Li et al. further showed that this NIR spectrum increases the lymphatic clearance of Aβ in the mouse brain, leading to cognitive improvements in AD models.^498^ Furthermore, Liu et al. in 2023 assessed the impact of tPBM on microglial cell function in diabetic mice and reported that tPBM stimulates the drainage system of the brain by activating mLVs, thus improving the effects of insulin treatment.^499^ Similarly, Wang et al. reported that NIR light therapy can enhance mLV endothelial cell function and mitochondrial metabolism, promote Aβ clearance, reduce neuroinflammation and neuronal damage, and improve cognitive function.^328^ Finally, in 2023, Oxana et al. discovered that a 7-day PBM treatment during deep sleep and wakefulness could better restore the clearance of Aβ from the brain and hippocampal ventricular system, suggesting that the influence of PBM of brain lymphatic vessels during sleep could provide a new foundation for research on sleep recovery function.^500^

The aforementioned groundbreaking studies revealed that the underlying mechanism involves NIR light enhancing mitochondrial respiration within LECs through photoreceptors such as CcO, repairing lymphatic endothelial junctions, and restoring the drainage function of mLVs, thereby facilitating the clearance of metabolic byproducts. Concurrently, NIR light can regulate the contractility of mLVs and promote lymphatic drainage through a mechanism mediated by NO. Despite these significant discoveries, gaps persist in our knowledge, and novel discoveries have attracted scholarly inquiry. A comprehensive understanding of the exact mechanisms by which NIR photobiomodulation modulates the cellular and molecular pathways within mLVs is needed. Additionally, the extant empirical evidence corroborating the therapeutic efficacy of NIR photobiomodulation in diverse animal disease models necessitates critical evaluation to ascertain its applicability in human clinical settings.

#### Magnetic stimulation modulation of mLVs

In 2023, Liu et al. explored the therapeutic potential of repetitive rTMS for ICH in a murine model. Research has revealed that rTMS has the capacity to modulate intracranial mLV drainage, potentially ameliorating ICH-induced neurological deficits. By utilizing CSF tracers, the team assessed metabolite clearance from the brain parenchyma after ICH and discovered that rTMS facilitated the recovery of this clearance function. These findings suggest that rTMS is a promising avenue for improving neurological outcomes following ICH by promoting mLV function.^501^

#### Sound modulation of mLVs

Recent studies have begun to reveal the intriguing interface between sensory stimulation and neurophysiological processes, with specific implications for neurodegenerative disease management. In their 2022 review, Sachdeva et al. meticulously examined the evidence linking music/sound, blood‒brain barrier permeability, and mLV clearance rates.^502^ Building on this exploration, Murdock et al. Subsequently advanced this area of research in 2024, where they explored the effects of 40 Hz multisensory audio‒visual stimulation on AD model animals. These findings indicate that such stimulation could increase mLV drainage, suggesting a novel therapeutic approach for neurodegenerative diseases.^503^ These investigations collectively highlight the potential of sensory stimulation as a method to influence neurophysiological processes, including the function of mLVs, and ameliorate disease symptoms. Nonetheless, critical questions remain, notably how these sensory modalities integrate at the molecular level to impact neuropathological states and whether these therapeutic strategies can be effectively translated from model organisms to human patients, paving the way for future investigations in this burgeoning field.

#### Electrical stimulation of mLVs

The exploration of noninvasive physical modalities, such as near-infrared, magnetic, sound, and electrical stimulation, has substantially enriched our understanding of mLVs and their function in neurophysiology. For example, Hauglund et al. in 2024 conducted a study where intracranial electrodes were implanted to observe the effects of chronic cranial electrical stimulation on lymphatic fluid transport. The findings indicated that the implantation of epidural electrodes led to reactive gliosis in the brain tissue and the cortex beneath the electrodes, as well as extensive mLV generation in the surrounding dura mater. This reactive gliosis was accompanied by an increase in the formation of mLVs, increased CSF lymphatic influx, and further reactive gliosis.^504^

#### Regulation of mLVs by continuous positive airway pressure

Breathing has been recognized to actively influence CSF flow within the brain, with implications for the homeostasis of CNS fluids. In a pivotal study conducted in 2023, Ozturk et al. explored the impact of assisted respiration through continuous positive airway pressure (CPAP) on lymphatic function in anesthetized, spontaneously breathing rodent models. These findings revealed that CPAP increased not only CSF flow velocity at the cranial base but also regional lymphatic transport. The increase in CSF flow velocity induced by CPAP was associated with an increase in ICP, including the amplitude of waveform pulsations.^505^ In summary, the study by Ozturk et al. provides compelling evidence that assisted breathing via CPAP can modulate CSF dynamics and lymphatic function, potentially offering a novel perspective on the management of CNS fluid homeostasis. The relationship between increased CSF flow and IICP, particularly in the context of waveform pulsations, warrants further investigation to fully understand the therapeutic implications of this intervention. However, the interplay between heightened CSF flow and the consequent rise in IICP, and how it relates to waveform pulsations remain areas for further exploration. Understanding the nuances of this relationship is essential to fully understand the therapeutic potential and implications of CPAP intervention for CNS fluid regulation.

To summarize, recent advancements in the fields of near-infrared, magnetic, sound, electrical stimulation and continuous positive airway pressure have deepened our understanding of mLVs. Increasing evidence suggests that noninvasive physical modalities possess significant potential in modulating mLVs, with promising results obtained in animal models. These findings pave the way for the development of novel therapeutic approaches for neurological conditions, leveraging the ability of noninvasive interventions to regulate the intricate mLV system. However, unresolved questions remain, such as the long-term effects of these modulations on the CNS and their translation into clinical practice for neurological disorders. Moreover, it is imperative to elucidate how these physical interventions can be optimized for safety and efficacy, offering new opportunities for innovative therapeutic strategies aimed at targeting the mLV system.

### Regulation of extracranial cervical lymphatics

The regulation of cervical lymphatics involves three approaches: pharmacological intervention, surgical methods, and physical manipulation. Pharmacological regulation of extracranial LVs involves agents such as adrenaline (an α-adrenergic ag onist) to modulate deep cervical lymphatic vessels and sodium nitroprusside to regulate deep cervical lymphatics, as well as the delivery of nanomaterials to the nasal passages and CLNs. Surgical approaches include the anastomosis of dCLNs with veins. Physical manipulation includes massage of the skull and CLNs to regulate mLVs.

#### Pharmacological modulation of extracranial cervical lymphatics

Targeting the regulation of central mLVs via extracranial lymphatics has been a recent research focus. Currently, pharmacological agents that modulate extracranial cervical lymphatics include adrenaline, nitroprusside, and the nasal and CLN delivery of nanomaterials.

##### Adrenergic (α-adrenergic agonist) regulation of cervical lymphatics

The study by Yoon et al. in 2024 represents a significant advancement in our understanding of lymphatic regulation. Their research revealed that low-dose phenylephrine (10 nM) increases TMR-dextran fluorescence in dCLNs by 51%, indicating increased CSF drainage. Similarly, the application of norepinephrine to cervical lymphatics increases drainage.^82^ These findings suggest that α-adrenergic agonists could be key in managing conditions associated with impaired lymphatic function. In essence, α-adrenergic modulation of cervical lymphatics, as evidenced by Yoon et al. represents a novel approach to bolster neuroimmune communication and treat lymphatic dysfunctions.

##### Sodium nitroprusside modulates extracranial cervical lymphatics

Sodium nitroprusside (SNP) is recognized for its vasodilatory effect on smooth muscle cells via NO release. The 2024 study by Yoon et al. revealed a novel aspect of SNP function, showing that low-dose SNP (3 μM) increases TMR-dextran fluorescence in dCLNs by 33%, indicating increased lymphatic drainage.^82^ In essence, the established vascular effects of SNPs are now complemented by their potential to improve lymphatic function, providing new therapeutic possibilities for managing lymphatic and neuroimmune disorders.

##### Liposomes enhance the mLVs delivery; CLN delivery

Recent advancements in drug delivery systems via mLVs offer an encouraging outlook for the treatment of brain diseases, particularly glioblastoma multiforme. In 2020, Zhao et al. demonstrated that the uptake of drugs in the brain was 44 times greater with the use of indocyanine green (ICG)-loaded PLGA nanoparticles administered through a subcutaneous (s.c.) injection at the neck near a local lymph node than with the intravenous injection route. This enhanced delivery enabled effective photodynamic therapy for the treatment of glioblastoma in mice.^222^ In addition, in 2022, Semyachkina-Glushkovskaya et al. reported the potential of liposomes for intranasal drug delivery to the brain, where they target glioblastoma multiforme.^218^ This study opens new avenues for improving the intranasal delivery of anticancer drugs via liposomes and noninvasive NIR laser technology. The findings of this research highlight the effectiveness of liposomes in crossing the nasal–brain barrier and delivering therapeutic agents directly to aggressive brain tumors. Together, these studies represent crucial advancements in the quest for noninvasive and precise drug delivery to the brain. However, questions remain regarding the optimization of these delivery systems for clinical use and their broader applicability across different types of brain pathologies. Further research is warranted to explore the long-term safety and effectiveness of these therapeutic stategies.

#### Cervical lymphaticovenular anastomosis (LVA)

Recent advancements in the treatment of neurodegenerative diseases have highlighted innovative surgical approaches that target meningeal lymphatic drainage pathways. In 2024, Xie et al. successfully performed microsurgical LVA on 50 patients with AD.^152^ Patients’ behavior, cognitive function, and memory are significantly improved as assessed by the Mini-Mental State Examination and the Montreal Cognitive Assessment.^152^ During the same period, Wu et al. reported the novel placement of vascularized submental LNs in the temporal subdural space in two patients diagnosed with symptomatic communicating hydrocephalus. At a minimum postoperative follow-up of 1 month, both patients experienced radiological and clinical improvements.^153^ Vascularized submental lymph node transfer and LVA are based on the latest theories of meningeal lymphatic drainage pathways. These procedures involve anastomosis and drainage between corresponding cervical lymphatic vessels and veins to reduce lymphatic pressure in deep brain tissues, accelerate lymphatic return from brain tissues, and clear the accumulation of metabolic products. Consequently, this process promotes CSF drainage, improves brain function, and facilitates the recovery of symptoms in individuals with neurodegenerative diseases such as AD and hydrocephalus.^152,153^ Despite these promising results, unresolved questions remain regarding the long-term efficacy and potential side effects of these procedures. Future research should also explore the scalability of these techniques to larger patient populations and their applicability across the spectrum of neurodegenerative disorders.

#### Physical modulation of extracranial cervical lymphatics

In recent years, noninvasive regulation of mLVs has emerged as a potential modality for treating CNS disorders. This section provides a comprehensive review of the latest advancements in the physical manipulation of extracranial cervical lymphatics and a reference for researchers in the field.

##### Regulation of mLVs by craniocervical manipulation

In a groundbreaking study conducted in 2022, Gao et al. reported that massaging the CLNs of patients with CSDH could facilitate the absorption of hematomas and increase the therapeutic efficacy of atorvastatin calcium.^506^ This innovative approach underscores the importance of physical modulation of extracranial lymphatics in enhancing the effects of medical treatments for CSDH. The implications of these findings are profound, suggesting that integrating lymphatic massage into standard care protocols could revolutionize the management of CSDH. Further investigation is warranted to fully elucidate the underlying mechanisms and refine this promising intervention for optimal patient benefit.

On the basis of the above discussion, current methods for modulating mLVs can be categorized according to the target site of action: modulation of mLVs, modulation of dCLNs, and modulation of the NPLP. Furthermore, these methods can be differentiated by the nature of the modulatory approach: noninvasive modulation (such as acoustic, optical, electrical, magnetic, and mechanical modulation), pharmacological modulation, and surgical intervention, among others (see Table 6 for details).

**Table 6:** Potential regulatory mechanisms of mLVs

Regulation of mLVs				
Property	Name	Parameter	Mechanism of action	Reference
Sound	Music	40 Hz	Promoting the drainage of mLVs	^502,503^
Photobiomodulation	Near-infrared	1000–2500 nm	Promotes the phagocytosis of microglia; regulates the microglial activation status; increase endothelial permeability; promote drainage by regulating vasodilation and constriction through NO; regulate LEC mitochondrial metabolism	^52,319,321,328,496,499^
Electricity	Chronic electroencephalography (EEG)	EEG signal: high-pass at 1 Hz and low-pass at 100 Hz; EMG signal: high-pass at 10 Hz and low-pass at 100 Hz	Provoking meningeal lymphangiogenesis, enhanced glymphatic influx of CSF, and reactive gliosis	^504^
Magnetism	Transcranial magnetic stimulation (rTMS)		Promoting intracranial lymphatic drainage	^501^
Force	Continuous positive airway pressure		Promoting mLV drainage by increasing intracranial pressure	^505^
Drug	VEGF-C		Promoting lymphatic endothelial cell proliferation; alleviating microglia-mediated inflammation and BDNF signaling in cells; promoting drainage	^28,39,62,167,250^
	Borneol		Improving mLVs drainage through mechanisms such as enhancing endothelial cell activity and promoting lymphatic vessel contraction	^128^
	Yoda1		Increasing intracranial pressure to promote CSF perfusion; activating VEGF-C	^493^
	Dobutamine		Improving blood perfusion and arterial pulsation in the brain	^53^
	Vitamin D		Protecting the structure of mLVs	^49^
	Yuanzhi powder		Restoration of AQP4 polarization	^494^
	DNase I		Degradation of NETs after IVH improves meningeal lymphatic system function and promotes endothelial activity restoration	^46^
Regulation of cervical lymphatics				
Physical	Manual		Increased negative pressure of the cervical lymphatics	^506^
Drug	Adrenaline		Promotion of contraction of cervical lymphatics	^78^
	Sodium nitroprusside		Increased dilation of cervical lymphatics	^78^
Surgery	Cervical lymphaticovenular anastomosis		Increased negative pressure of the cervical lymphatics	^152,153^
Regulation of the nasopharyngeal lymphatic plexus				
Biological materials	Liposomes and near-infrared laser		Material delivery of drugs	^218^

## Conclusions and outstanding questions

The emerging evidence concerning mLVs, summarized above, highlights the critical involvement of mLVs in both physiological homeostasis and pathological processes. Research advancements have lent support to the notion that the distribution of mLVs extends far beyond what is currently recognized and that a glymphatic system within the brain parenchyma is directly connected to mLVs in the CAV, whereas the CSF in the subarachnoid space communicates directly with mLVs through LECs in the AG and ACE, participating in a multitude of roles involving CSF, metabolic waste, senescent cells, and various functions within central and peripheral immune activities. However, several key issues need to be addressed before these new concepts can be translated into meaningful therapeutic strategies.

Currently, our understanding of the developmental mechanisms, structure and distribution of mLVs and their associations with their functions remain unclear. Although there is yet no evidence to suggest that mLVs develop from peripheral lymphatic vessels, cranial mLVs develop postnatally rather than during the embryonic stage.^75^ The VEGFC‒VEGFR3 signaling axis is a crucial pathway for the development of mLVs, where the absence of VEGFC leads to the dysfunction of mLVs, and supplementation with VEGFC improves their drainage ability. What remains to be clarified is whether exogenous or excess VEGFC can result in incomplete, immature, and abnormal formation of mLVs.^252^ For example, studies have shown that although there is proliferation of dorsal mLVs in a mouse model of viral meningitis, their drainage function is impaired.^62^ Furthermore, in models of IVH, lymphatic thrombi within mLVs may lead to drainage dysfunction, whereas dysfunction in basal mLVs, which are central to the drainage of CSF and the downstream region of dorsal mLVs, may cause compensatory proliferation in dorsal mLVs.^46^ Although VEGFA and VEGFR2 play a role in the development of peripheral lymphatic vessels^162^ and VEGFR2 is also a key molecule in the formation of corneal lymphatic vessels,^161^ VEGFA’s regulatory effect on the development of mLVs remains to be elucidated. It remains unclear whether dorsal mLVs participate in broad immunoregulation due to their immature structural development and whether their zipper-like junctions facilitate the entry of immune cells. Similarly, there are outstanding questions regarding whether basal mLVs, potentially due to their mature morphology, larger diameters, button-like connections, and presence of lymphatic valves, act as CSF drainage hubs. Further investigations are needed to determine the specific role of the basal mLVs adjacent to cranial foramina in CSF drainage and immune functions, as well as the role of mLVs in mediating drainage through the cribriform plate.^21^ An enigma yet to be resolved concerns the proportion of CSF drainage attributed to the skull LVs in the dura mater and their role in immunological processes. Recent studies have identified LYVE-1+ cells in both the AG^87^and the ACE,^79^ suggesting that CSF and metabolic byproducts may directly enter mLVs through ACE or the AG. However, these concepts require further investigation. mLVs distant from the venous sinuses have been classified into three types,^84^ However it is currently unclear whether the expression of these lymphatic vessel types represents distinct structural forms of mLVs and whether they perform different functions. The existence of mLVs within the mammalian brain parenchyma is still controversial^197^ and requires additional research for clarification. An intriguing question remains regarding the precise proportion of CSF drainage caused by the newly identified NPLP,^82^ which is not yet understood. Furthermore, the structure of CAV mLVs^80^ and their role in connecting cerebrovascular lymphatics and mLVs, as well as the relationship between the ocular lymphatic system^89^ and mLVs, are aspects that require further investigation.

In the study of the function and mechanisms of mLVs, there are still numerous mysteries regarding the metabolites they drain, their interactions with different cell types, and their role under pathological conditions. Metabolic byproducts drained by mLVs include Aβ,^28^ tau proteins,^40^ α-syn,^38^ TDP-43,^33^ and glutamate neuronal debris, among others. However, the mechanisms by which these metabolites enter mLVs are not fully understood. A critical question is whether there is an upper limit to the molecular weight and size of these substances, which is crucial for the design of mLV-targeted drug delivery systems. Furthermore, the detailed molecular processes involved in the drainage of senescent cells, including astrocytes^36^ and red blood cells,^54^ through mLVs remain largely enigmatic. How DCs,^132^ B cells,^188^ T cells,^17,190^ and neutrophils^46^ enter and migrate out of the CNS via mLVs under both homeostatic and CNS disease states, as well as how the periphery influences CNS immunity through the mLV‒CLN axis,^208,209^ cribriform plate,^216,217^ eye,^89^ and bone marrow,^91,188^ are questions that require further research and clarification. This includes exploring the mechanisms of intranasal^218^ and CLN^221^ drug delivery and examining complex ocular–brain and cranial–immune interactions in detail. It remains unclear whether the VEGF-C-VEGFR3 signaling pathway is affected during the aging process^78,219^ and whether reversing age-related alterations in mLV functions in a targeted manner is possible. The pressing questions that need to be addressed include the following: Can therapeutic approaches directed at mLVs effectively suppress or reverse neurodegenerative changes in humans? What are the long-term effects of such treatments? How can the most effective therapeutic strategies be personalized for each patient? Additionally, Chen et al. reported that an increase in mLV drainage was associated with SAH after subarachnoid hemorrhage^174^; however, these findings are currently controversial. Studies suggest that blockade of CLNs may reduce intracranial inflammation caused by MCAO^48,209,211^; however this discovery is controversial, as ligation of the dCLNs is generally known to exacerbate intracranial inflammation.^54^ How bacterial infections other than sepsis and LM proliferation on mLVs are still not clear. How the metabolic byproducts released by red blood cells after cerebral hemorrhage can trigger the activation of the central immune system is another interesting area of research, including exploring how inflammation storms could be controlled or central effects mitigated through mLVs, as well as peripheral consequences or effects on the central system. CSF permeates into the cranial bone marrow through dural channels, where it influences a variety of cells in the bone marrow microenvironment.^91^ Although our research team detected LYVE-1+ cells within the cranial vault, further studies are needed to determine whether lymphatic vessels exist within the skull.

Noninvasive observation of human mLVs and in vivo observation in animals represent future critical areas of research. Owing to the continued development and maturation of noninvasive observation technologies, we anticipate that a series of mysteries regarding the drainage of CSF through mLVs will be unraveled. Of particular importance is revealing whether the primary CSF drainage points in human mLVs are located above or posterior to the superior sagittal sinus^80^ and whether dural channels serve as auxiliary pathways for CSF efflux. In addition, some clinically relevant issues are explored. For example, whether scalp massage could improve treatment outcomes in the management of chronic subdural hematoma, similar to the effects of massage on cervical lymph nodes, is unclear. Additionally, the issue of sex differences that necessitate further investigation, specifically why males exhibit a greater CSF drainage capacity than females do,^80^ is worthy of attention. The variability in CSF drainage through mLVs across different disease models is another area of concern worth monitoring. NIR^123,137^ imaging and photoacoustic imaging techniques^145^ have been successfully used to observe dynamic drainage in mouse mLVs, although these techniques have not yet been widely reported in human mLV studies. Therefore, NIR and photoacoustic imaging are promising noninvasive approaches for investigating human mLVs in the future. Currently, owing to experimental constraints, ex vivo tissue staining remains the mainstream method for studying mLVs, especially for dorsal mLVs, which are more easily accessible. With technological advancements, more studies on basal mLVs and the NPLP are anticipated to emerge. Pending questions, such as the distinct features of CAV mLVs and the exact mechanisms by which they interact with the glymphatic system, are expected to be progressively addressed. A thorough understanding of the pathways involved in the transport of CSF and macromolecules from the brain to mLVs will enhance our understanding of the importance of CSF drainage mechanisms and neur-immune interactions under physiological and pathological conditions, and will aid in the development of novel delivery systems for therapeutic agents targeting the brain.

The injury and regulatory molecular mechanisms of mLVs under CNS disease conditions will be a research focus for the next decade. Studies on the injury mechanisms involving mLVs generally revolve around the development of LECs and lymphatic valves, with the VEGFC–VEGFR3 signaling pathway at the core. On the basis of current research on peripheral lymphatic vessel injury mechanisms,^162^ genes such as Sox18, Fat4, ADAMTS3, FBXL7, GJC2, PTPN14, KIF11, ITGA9, REELIN, EPHB4, and CALCRL may be potential targets for investigating the injury mechanisms of mLVs. Research will further elucidate many mysteries that are currently unsolved. For example, studies have shown that VEGFC can regulate mLV dysfunction caused by toxoplasmosis, yet it is unable to improve brain edema,^57^ a fascinating phenomenon that urgently requires in-depth analysis. The molecular signaling pathways associated with the development or regulation of lymphatic valves remain largely unclear,^266^ particularly the specific mechanisms of interaction between GATA2 and Prox1, as well as the regulatory elements that modulate the influence of shear stress on GATA2 expression.^266^ In addition, the upstream regulatory relationships between GATA2 and PROX1 are still not well defined.^257,267^ In addition to Prox1, a variety of shared molecular pathways impact the development of both lymphatic and venous valves. An increase in intracranial pressure is commonly observed in various diseases such as hydrocephalus, TBI, ICH, IVH, and posttumor complications. Concurrently, reductions in mLV drainage have been observed in models of these diseases; however the potential link to the regulation of the Piezo1 pathway has still not been conclusively determined. It is unclear whether the reduced clearance rate during sleep is associated with blood pressure changes and Piezo1-mediated mLV drainage. It remains a mystery whether there is a threshold range for the Piezo1 pathway, whereby excessive intracranial pressure or reduced blood pressure to a certain threshold could result in diminished mLV drainage. Additionally, the specific relationships between sleep disturbances and mLV drainage or the Piezo1 pathway require further clarification. Although previous studies have indicated that ERK1/2 act as upstream regulators of SOX18^290^ and defects in SOX18 cause HLTS, the exact relationship between SOX18 and the injury mechanism of mLVs is still unclear. Furthermore, elderly mice exhibit low expression levels of vascular endothelial cadherin in mLVs,^78^ whereas models of migraines have shown that an increase in VE-cadherin expression in mLVs is mediated by the CGRP-CLR signaling pathway.^316^ The elevation of VE-cadherin in different models is currently a matter of debate regarding whether it represents mLV dysfunction or recovery. Presently, pharmacologically regulated mLVs primarily target the VEGFC-VEGFR3 signaling pathway, with studies attempting to use molecules such as vitamin D and Yoda1 to repair connections between LECs and thereby regulate mLV permeability. Strategies for modulating mLVs can be categorized by target: the mLVs themselves, the cerical lymphatics, and the NPLP. Through interdisciplinary cooperation, noninvasive treatments, including sound,^502,503^ light,^52,319,321,328,496,499^ electricity,^504^ magnetism,^501^ and mechanical forces,^505^ as well as pharmacological interventions,^28,39,49,53,62,128,250,493,494^ have made progress. Preliminary studies using liposome delivery of VEGFC have increased the functionality, and NIR has been effectively employed to regulate mLV drainage, whereas acoustic and electrical stimuli, and CLN massage have shown potential therapeutic effects. However, most studies are in the early stages regarding the mechanism and evidence level, necessitating increased clinical involvement. While reports indicate that LVA may be significantly effective for treating Alzheimer’s disease and hydrocephalus,^152,153^ these findings require further validation through high-quality studies because of the small sample sizes and limited number of research centers. Advances in noninvasive and surgical intervention techniques, along with progress in materials science, will further deepen the understanding of mLVs in immunological research. Current discoveries of drug administration to dCLNs^221^ and LVA^152,153^ have achieved preliminary success in treating brain tumors, dementia, and PD. Notably, the discovery of the “eye‒brain‒immune” axis^89^ has shifted the perception of brain immunity from a region of “immune privilege” to one of “unique immunity”.^507,508^

To summarize, a concerted effort should be undertaken as follows: 1). The mechanisms underlying the development of mLVs and the relationships between the structural diversity of mLVs and their functions should be ascertained. 2). The precise pathways through which CSF, metabolic byproducts, senescent cells, and immune cells enter mLVs should be identified. 3). Understand the potential molecular mechanisms of mLV damage and regulation in the context of CNS disease states. 4). The characteristics of potential molecular materials that can traverse mLVs should be determined to develop effective single or combination therapeutic modalities that may regulate CNS disease progression through mLVs. 5). Furthermore, in animal models, the potential mechanisms by which acoustic, photonic, electrical, and magnetic modalities can modulate mLVs should be elucidated since such methods hold broader prospects for clinical translation. A deeper understanding of the mLVs will facilitate a more complex understanding of brain immunity and offer a wealth of new opportunities for designing innovative therapeutic interventions and drug delivery strategies, thereby expanding the range of potential molecular and cellular targets for disease-modifying therapies.
